# Availability of essential medicines in Pakistan—A comprehensive document analysis

**DOI:** 10.1371/journal.pone.0253880

**Published:** 2021-07-09

**Authors:** Sunaina Rafi, Huma Rasheed, Muhammad Usman, Hafiz Awais Nawaz, Syed Muneeb Anjum, Mamoona Chaudhry, Zaheer-Ud-Din Babar

**Affiliations:** 1 Institute of Pharmaceutical Sciences, University of Veterinary and Animal Sciences, Lahore, Pakistan; 2 Department of Epidemiology and Public Health, University of Veterinary and Animal Sciences, Lahore, Pakistan; 3 Centre for Pharmaceutical Policy and Practice Research, University of Huddersfield, Huddersfield, United Kingdom; Xiamen University - Malaysia Campus: Xiamen University - Malaysia, MALAYSIA

## Abstract

**Introduction:**

Access to essential medicines (EMs) is a basic human right. Non-availability and shortages of EMs are reported for Pakistan but there is insufficient data to define the nature and magnitude of this problem. The current study is designed to systematically analyze the medicines included in the National Essential Medicines List (NEML) for their availability through comprehensive document analysis.

**Methods:**

An expanded list of medicinal items was developed using the NEML of Pakistan (2018) to enlist individual medicines with their specifications. Registration status of the medicines was searched using three publicly accessible information sources; Pharmaguide 25^th^ Edition, 2018–19, the on-line Drug Information System, and the Mobile Application Pharmapedia followed by a later 3-step validation of the data. The unregistered EMs were then further categorized into three subgroups in accordance with their possible remedial strategies.

**Findings:**

The 19 studied categories comprised 690 EMs and it was found that 179 (26%) of these EMs don not have a registration status. However, it was also identified that the availability of 47 (26.2%) out of 179 unregistered EMs can be enssured by strengthening compounding services, and prioritizing registration of age-appropriate formulations. Availability of another 39 (21.7%) such medicines can be ensured by revising the NEML or the product registrations for the slight differences in their different specifications. The categories showing high proportion of unregistered medicines included anti-Parkinson’s medicines (100%), antidotes and other substances used in poisoning (60%), diuretics (47%), anticonvulsants/antiepileptics (42%), hormones and other endocrine medicines and contraceptives (38%), medicines for mental and behavioral disorders (30%), anti-infectives (27%), medicines for pain and palliative care (26%), medicines for neonatal care (25%), medicines for diseases of joint (25%), gastrointestinal medicines (24%) and cardiovascular medicines (15%).

**Conclusion:**

The study shows the absence of registration status of a significant number of EMs in Pakistan. This could be major barrier in their access. Strategies are needed to strengthen the processes of their registration on priority basis.

## Introduction

Essential medicines (EMs) fulfill the priority health care needs of majority of population, and are required to be available in adequate amount in suitable dosage form at all times [1]. EMs policies are also integrated to encourage Universal Health Coverage (UHC) and play a major role in the accomplishment of sustainable development goals [2]. In 1977, the first model list of 212 EMs was published by the World Health Organization (WHO) and in 1978, EMs were included as an 8^th^ component of primary health care in the Alma Ata Declaration [2]. Poor availability of EMs had caused disability, illness, and ultimately deaths of millions of people around the world. Absence of treatment, poor treatment and high possibility of medication errors could be due to the undesirable compensatory approaches used as alternates for ‘missing EMs’ [3]. Guan *et al*. has quoted a WHO survey conducted in 2013 which estimated that an active National Essential Medicines Policy (NEMP) can prevent more than 10 million deaths per year worldwide [4]. Global Action Plan for prevention and control of non-communicable diseases (NCDs) has set up a voluntary target of 80% availability for EMs used in the treatment of NCDs by 2025 [5]. According to WHO and Health Action International (HAI), the availability of EMs below 50% is graded as low whereas a range of 50–80% availability is considered fairly high [6]. Even though many Low- and Middle-Income Countries (LMICs) have National Essential Medicines List (NEMLs), still 50% of the world’s population do not have access to EMs [4, 7].

WHO has also developed a model list of EMs for children and currently 7^th^ Edition has been updated in 2019 [8]. In 1994, Pakistan developed its first National Essential Medicines List (NEMLPK) with subsequent renewals in 1995, 2000, 2003 and 2007 [9]. In 2013, Pharmacy Services Division under the newly founded Drug Regulatory Authority of Pakistan (DRAP) prepared the essential medicine list based on health conditions, disease burden and affordability. The NEMLPK was revised in 2016 and 2018 containing 415 and 428 medicines, respectively. This was based on 19^th^ and 20^th^ WHO model lists of Essential Medicines (WHOEMLs). These revisions were made through a consultative process involving multidisciplinary health care professionals as per the guidance of WHO [9]. The NEMLPK also provides information on the facility level (primary, secondary and tertiary) at which the essential medicines are recommended for use. The use of NEML and formularies vary significantly across the facilities as observed in a study across the city of Bhawalpur District [10]. In 2020, a notification was issued by Ministry of National Health Services, Regulation and Coordination, Government of Pakistan which declared the adoption of current WHOEML for the purpose of drug pricing policy in Pakistan [11]. However, the official mobile application for NEML by DRAP [12] still includes the 2018 revision of NEML as the last updated version. WHO strategies for overcoming global shortages recommend development of consolidated lists of EMs that are short in supply or are at risk of short supply [3].

At earliest the issue of availability of EMs was highlighted in the regular reports published by Network of Consumer Protection in Pakistan [13]. According to one of these reports, 11 key drugs were missing from the market in 2001 including diuretics, anti-anginal drugs, poisoning antidotes, immunosuppressants, anti-hypertensive and anti-fungal agents [13]. Later in 2003 and 2005, Rasheed H *et al*. conducted a study on the dilemma of Missing Essential Drugs of Pakistan, by checking the market availability as well as the registration status of essential and clinically important medicines followed by the interviews from the clinicians on the clinical impact of shortages and non-availability of these medicines [14]. The government documents, press, as well as scientific publications [15] also stated phenytoin, thiazides, adrenaline, thyroxine [16], primaquine, and folic acid [17] as missing EMs. Many of these medicines were not being manufactured by stakeholders and drugs manufacturers due to less profits [15]. In Pakistan the term Orphan Drug has been used as a misnomer and quoted in publications to represent the missing and non-available essential medicine [15], which is not to be encouraged.

Hence, some of the terms used in this manuscript are described in Box 1 for clarity.

**Box 1. Important definitions**

**Generic name of essential medicine**

Each essential medicine is described in this manuscript with its generic name as used in the WHOEML or NEMLPK-2018.

**Expanded list of essential medicines**

Every generic in the essential medicine list is expanded to describe its full specifications including dosage and strength along with any other special specification. The resultant list of essential medicines is described as expanded list of essential medicines in this manuscript.

**Registration of medicines and market authorization**

Authorization of a medicine by the national drug regulatory authority (NDMA) for manufacturing, import, sales and distribution is termed registration of medicine (as practiced locally in Pakistan) or provision of market authorization (as practically globally in many countries). The medicines hence registered with the NDMA are termed registered medicines. The medicines which are not registered are classed as ‘unregistered medicines’.

**Non-available or Missing Essential Medicines**

Non-available or Missing Essential Medicines are the terms used to describe the absence of essential medicines from the market supply. The reasons for the non-availability of essential medicines include lack of interest from manufacturers and importers due to less profitability, global shortages, poor supply chain management as well as less demand from the consumers and prescribers. This could be because of lack of compliance to Standard Treatment Guidelines.

**Orphan Drugs/Medicines**

As per US FDA Orphan Medicines is defined as the “Drugs (includes biologics) for the prevention, diagnosis, or treatment of diseases or conditions affecting fewer than 200,000 persons in the US OR–Drugs that will not be profitable within 7 years following approval by the FDA(1).

There were gaps in evidence regarding availability of EMs in Pakistan [15]. Most published literature includes market surveys, based on the selected list of medicines predominantly including the medicines with market authorization. Hence the documentation of the medicines lacking market authorization were completely neglected and faced the dilemma of staying in void. A comprehensive study with systematic methodology was needed in this context to assess the national situation with respect to the availability of EMs. Evidence produced from the study can then be used to define the magnitude of the problem as well as to guide the development of evidence-based medicines policies. Due to technical and ethical reasons only the medicines with registration status can be taken to the floors/drug sale outlets for the survey on physical availability. Hence, the primary objective of this study was to carry out a systematic analysis of NEML using public documents and information sources to evaluate the registration status of EMs in Pakistan as a first step to carry out comprehensive study on availability of essential medicines in the country. Another objective was to develop recommendations and suggestions on the measures needed to improve the availability of EMs in the country.

## Methods

National Essential Medicines List of Pakistan (NEMLPK-2018), 7^th^ Edition, 2018 [18] was analyzed using three publicly available information sources; Pharmaguide, 25^th^ Edition, 2018–19 [19], the on-line Drug Information System [20] and the Mobile Application Pharmapedia [21] to attain information on the registration status and to confirm the specifications of items enlisted in NEML. The Management Information System (MIS) database from the Drug Regulatory Authority of Pakistan (DRAP) website [22] was used for the validation of registration status of medicines. WHO Model List of EMs, 20^th^ Edition, 2017 [23] was used for comparison with NEMLPK-2018. A data collection form was developed to extract information from above mentioned sources regarding registration and specifications of EMs in NEMLPK-2018 (S1 File). A comprehensive procedure for document analysis was carried out adapting the guidelines provided in literature [24].

### Step one: Expansion and allotment of Unique Identification Numbers (UIDs)

**Fig 1:**
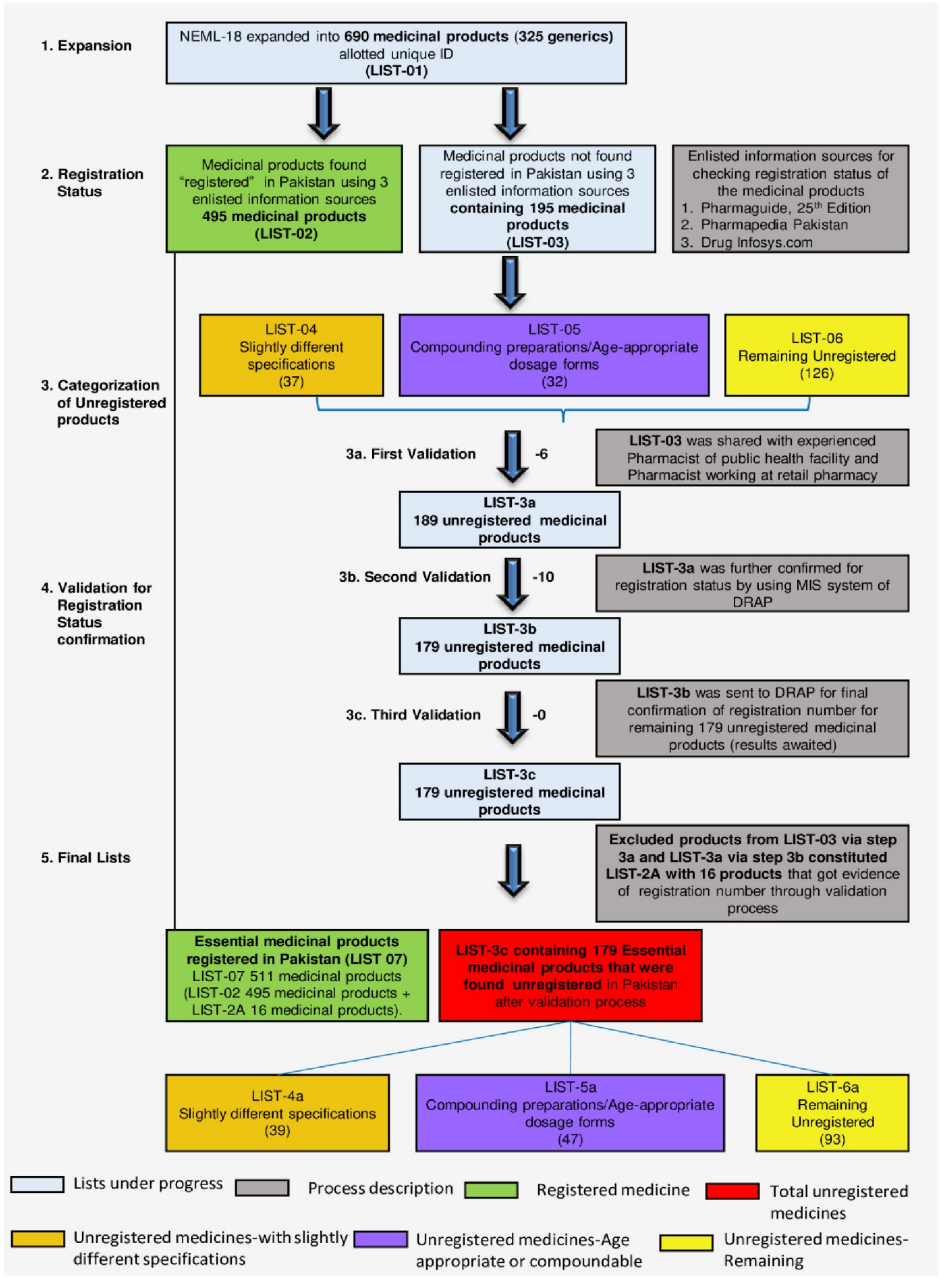
An attrition chart showing NEMLPK-2018 edition processed to various stages including expansion to medicines, confirmation of registration status and validation steps.

The generics mentioned under each category of NEMLPK-2018 were carefully detailed to yield “List of expanded medicines” (LIST-01) with specification covering dosage form, strength, and any special specification (e.g., scored, chewable tablet allotting a unique ID number for each medicine. This list was processed as described in Fig 1 to generate a comprehensive data of medicines which hold registration status in Pakistan. For example, the solid oral dosage form (capsule) for omeprazole had three different specifications 10 mg, 20 mg and 40 mg. So for each specification a different UID has been allotted).

### Step two: Registration status information collection

Registration status of each medicine in LIST-01 was confirmed using the three information sources mentioned above. Any medicine that was found registered using any one of these information sources was categorized as “Registered” medicines in Pakistan (LIST-02). The medicines not found in information sources were considered as “Unregistered” medicines in Pakistan (LIST-03). These lists were given green and red color codes, respectively.

### Step three: Analysis of the generated lists of EMs

LIST-03 of unregistered medicines (Red) was further analyzed for classification as medicines which are “Registered but with slightly different specifications LIST-04 (orange)” and “Medicines that can be prepared through compounding or were included as age-appropriate preparations LIST-05 (purple)” and “Remaining medicines without registration status LIST-06 (yellow)”. Color coding was used for convenience in summarizing the results and clarity during data handling.

### Step four: Registration confirmation through 3-step validation

The LIST-03 (including LISTs 04–06) were validated through a three-step validation process. Firstly, by sharing with two experienced pharmacists working in public sector hospital and at retail pharmacy to identify any item that is registered in Pakistan and their classification in the orange, purple and yellow categories. Brand name, manufacturer/company name and registration numbers of the medicines were noted as an evidence for registration. The medicines still lacking information on registration were titled as LIST-3a and were validated for registration status by using DRAP MIS system (Provisional List of Registered Medicines) [22] as a second step of validation. The resultant list of unregistered medicines LIST-3b was shared with DRAP for final confirmation of registration status.

### Data analysis

Impact of unregistered status on a particular NEML category was determined by calculating the percentage of medicines lacking registration status for each studied category. This was calculated by using simple descriptive statistics including the following formula:

$$\begin{array}{l} \text{The}\;\text{percentage}\;\text{of}\;\text{unregistered}\;\text{medicines}\;(\%)=(\text{Number}\;\text{of}\;\text{Medicines}\;\text{in}\;\text{the}\;\text{NEML}\;\text{category}\;“\text{Z}”\;\text{that}\;\text{lacked}\\ \text{proof}\;\text{of}\;\text{registration}/\text{Number}\;\text{of}\;\text{essential}\;\text{medicines}\;\text{in}\;\text{expanded}\;\text{list}\;\text{of}\;\text{medicines}\;\text{in}\;\text{the}\;\text{NEML}\;\text{category}\;“\text{Z}”)\;\text{x}\;100 \end{array}$$

## Results

The primary document of NEMLPK-2018 contains 30 basic categories of medicines used for different clinical conditions. In the current study 19 out of 30 therapeutic categories of NEML were analyzed for their registration status and were processed stepwise as illustrated in the Fig 1. Total 19 therapeutic categories of NEML of Pakistan, 2018 Edition (NEMLPK-18) were expanded to yield LIST-01 titled “Expanded List of medicines from 19 categories”. This list contained 325 generics with 690 medicines (S2 File) corrected to 644 after identification of 46 medicines that appeared more than once (S3 File). The list of 690 medicines was then evaluated to check the registration status of medicines using the three information sources mentioned earlier (Fig 1).

**Fig 2:**
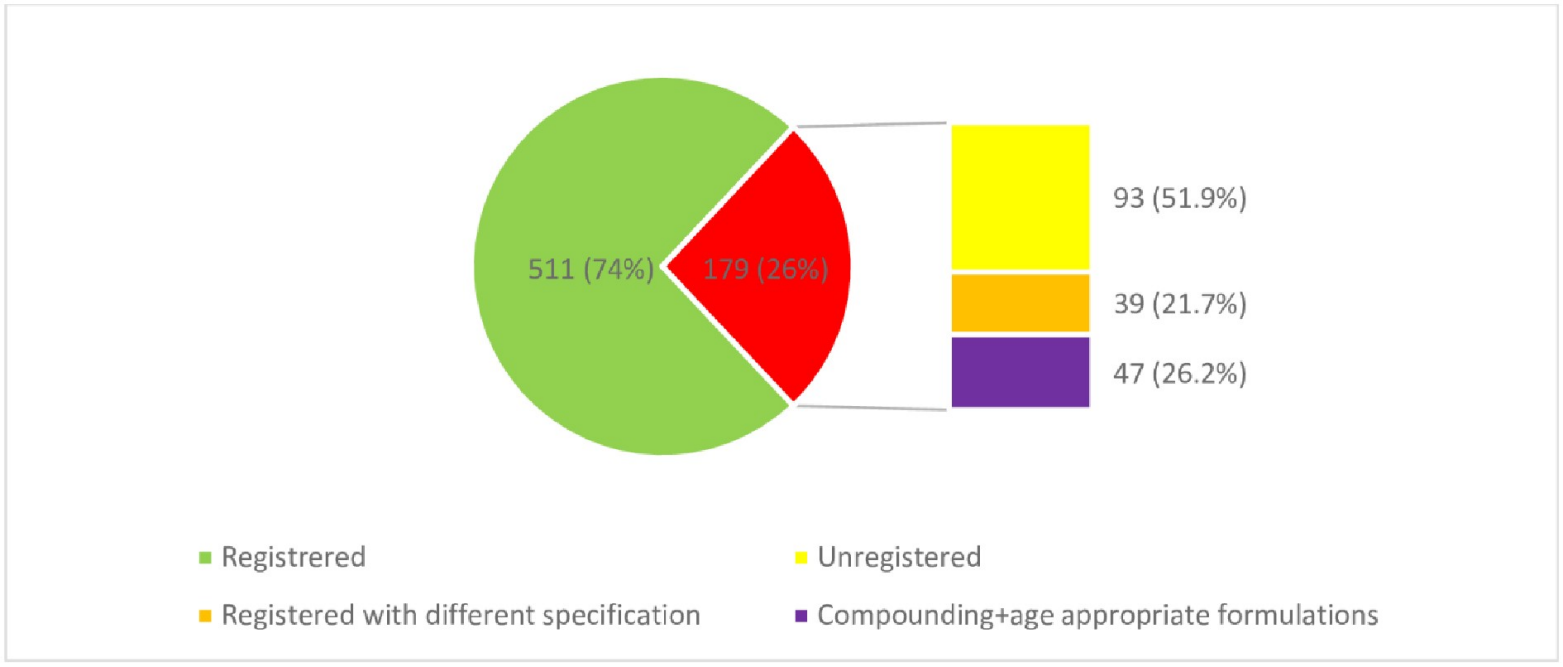
Distribution of 690 medicines according to registration status and color coding.

**Fig 3:**
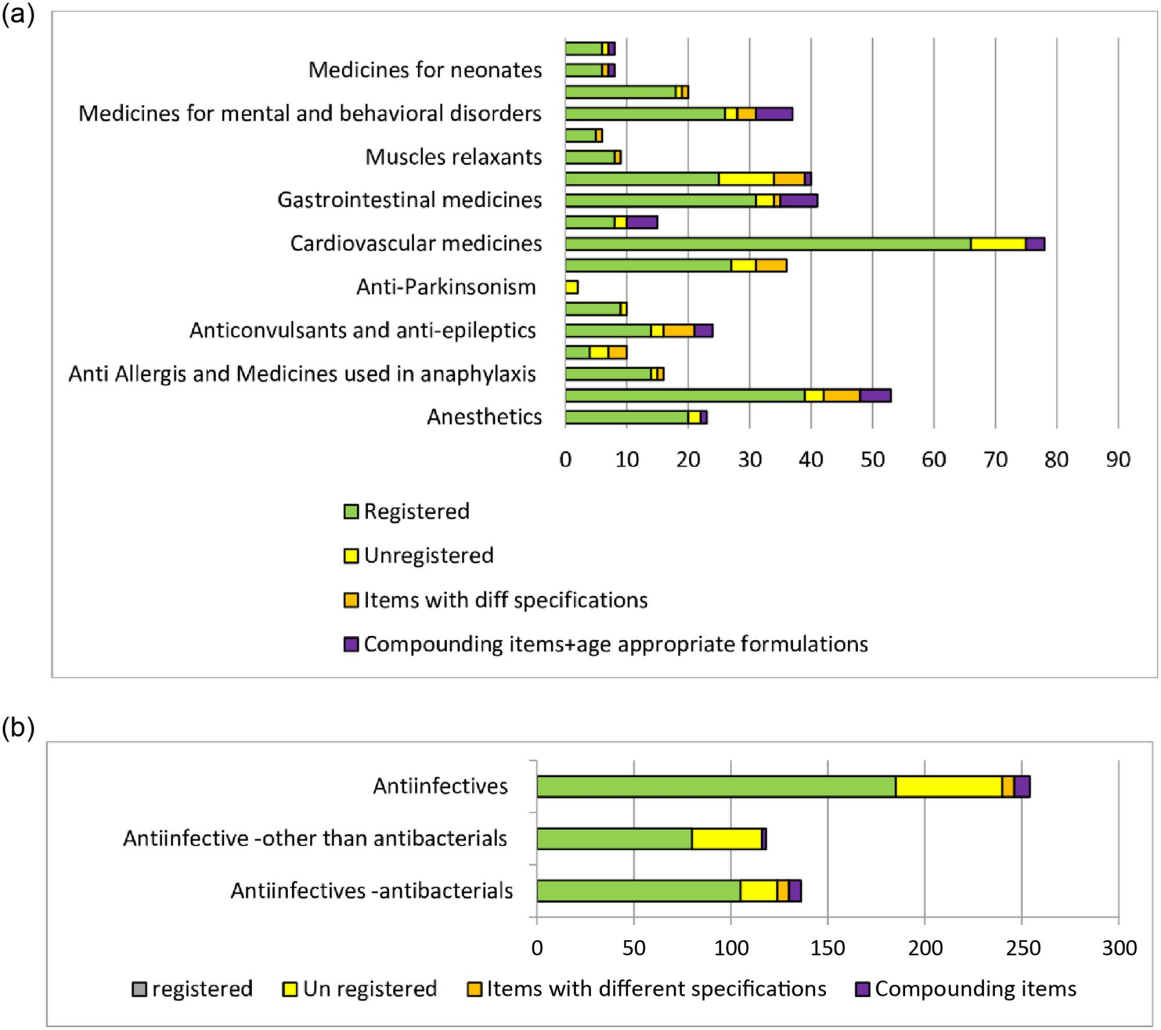
**a**. Distribution of 19 categories of medicines (N = 690) in NEML according to their registration status and color coding. **b**. Distribution of 254 anti-infectives in NEML according to their registration status and color coding.

Table 1 shows the summary of medicines and therapeutics categories enlisted in NEMLPK-2018 that were analyzed in the current study. Summary of descriptive data of the 19 studied therapeutic categories is described in Table 3 with the total numbers for generic, expanded medicines, categorized as “registered essential medicines (LIST-07: green)” and “un-registered essential medicines (LIST-3c: red)”. As the purpose of current study includes identification of possible solutions for ensuring availability of items facing availability issues, the latter category of unregistered medicines is further screened for the medicines as “List of medicines that are registered with slightly different specifications (LIST-4a: orange)” and “List of medicines that can be prepared through compounding or are age-appropriate preparations (LIST-5a: purple)” and “List of remaining unregistered medicines (LIST-6a: yellow)”. Fig 2 shows the distribution of total 690 medicines in accordance with their registration status. Fig 3a and 3b shows the category wise distribution using the color-coding scheme mentioned above. The descriptive statistics on registration status are shown in Table 3.

**Table 1:** Summary of therapeutics categories enlisted in NEMLPK-2018 analyzed in the study.

Attribute studied	Number/description
No. of categories analyzed from NEML 2018	19
Total number of generics in the analyzed categories	325
Total number of medicines found in the analyzed categories	690–46* = 644

* Number of duplications identified = 46

The stepwise generation of the analysis of NEMLPK-2018 is described below:

### Step one: Expansion and allotment of Unique Identification Numbers (UIDs) (LIST-01)

The 19 categories of NEMLPK-2018 were expanded to generate 395 generics into 690 individual medicines which were identified using Unique Identification Codes (UIDs) to ensure traceability of data and avoid duplications. LIST-01 titled as “Expanded list of Essential Medicines for Pakistan” according to the NEMLPK-2018 (S2 File) including the status of its inclusion as primary (P), secondary and tertiary care (T) status.

### Step two: Registration status “provisional lists of essential medicines registered in Pakistan (LIST-02)” and “provisional list of unregistered essential medicines in Pakistan (LIST-03)”

Using the three information sources 495 medicines were found to be having registration status in Pakistan constituting LIST-02 and the 195 medicines lacking information on registration was grouped in LIST-03 of unregistered medicines. A complete flow of development of different lists in the study originating from NEMLPK-2018 is described in Fig 1.

### Step three: Analysis of the generated lists of essential medicine for categorization

LIST-03 was analyzed, and color coding was used for convenience in summarizing the results and clarity. Provisional list of EMs was generated. This included the “LIST-04 containing 37 medicines registered but with slightly different specifications (orange)” and “LIST-05 containing 32 items which were identified as medicines that can be prepared through compounding or were included as age-appropriate preparations (purple)” and “LIST-06 containing 126 remaining medicines without registration status (yellow)”.

### Step four: 3-step validation

A process of validation was conducted as laid down in methods section which showed following results to confirm the registration status as well as for their categorization in and analysis of the list of 195 medicines (LIST-03) for which no information was accessible through the information sources used. Results of validation are summarized in Table 2.

**Table 2:** Results of 3-step validation.

UID	Medicines	Coding/Status and registration details	
		Before validation	After validation
	**First Validation (confirmation from Senior Pharmacists in retail and public sector through provision)**		
	LIST-03 = 195 medicines		
	(37 orange (LIST-04), 32 purple (LIST-05) and remaining 126 yellow (LIST-06)		
UID-01	Inhalational isoflurane	Yellow	Green
UID-02	inhalational sevoflurane	Yellow	Green
UID-98	calcium gluconate injection 100mg/ml in 10ml ampoule	Yellow	Green
UID-104	carbamazepine tablet (chewable) 100mg	Yellow	Purple
UID-105	carbamazepine tablet (chewable) 200mg	Yellow	Purple
UID-121	phenytoin tablet (chewable) 50mg	Yellow	Purple
UID-247	pyrazinamide tablet (dispersible) 150mg	Yellow	Purple
UID-248	pyrazinamide tablet (scored) 150mg	Yellow	Purple
UID-291	nystatin lozenges 100,000 IU	Yellow	Purple
UID-302	abacavir syrup 100mg/5ml	Yellow	Purple
UID-323	ritonavir syrup 400mg/5ml	Yellow	Purple
UID-366	artesunate rectal dosage form 50mg	Yellow	Purple
UID-367	artesunate rectal dosage form 200mg	Yellow	Purple
UID-559	glycerin suppositories (previously categorized in compounding list)	Purple	Green
UID-607	suxamethonium powder for injection	Yellow	Green
UID-647	nicotine replacement therapy chewing gum 2mg	Yellow	Purple
UID-648	nicotine replacement therapy chewing gum 4mg	Yellow	Purple
UID-651	methadone concentrate 5mg/ml	Yellow	Purple
UID-652	methadone concentrate 5mg/ml	Yellow	Purple
UID-653	methadone oral solution 5mg/5ml	Yellow	Purple
UID-654	methadone oral solution 10mg/5ml	Yellow	Purple
UID-682	surfactant suspension for intratracheal instillation 25mg/ml or 80mg/ml	Yellow	Green
	**Second Validation (DRAP-MIS data)**		
	LIS3a = 189		
	(104 yellow, 38 orange and 47 purple)		
UID-221	sulphamethoxazole + trimethoprim 80mg + 16mg/ml in 10ml ampoule	Yellow	Green
UID-262	ethionamide tab 125mg	Yellow	Green
UID-463	enalapril tablet 2.5mg	Yellow	Green
UID-482	enalapril tablet 2.5mg	Yellow	Green
UID 588	insulin injection (soluble) 40 IU/ml in 10ml vial -	Yellow	Green
UID-590	intermediate acting insulin injection 40 IU/ml in 10ml vial	Yellow	Green
UID-15	lidocaine + epinephrine (adrenaline) injection 2% hydrochloride + epinephrine 1:80 000	Orange	Green
UID-361	meglumine antimoniate injection 30%, equivalent to approximately 8.1% antimony in 5ml ampoule	Orange	Green
UID-384	paracetamol oral liquid 125mg/5ml	Orange	Green
UID-454	adenosine injection 3mg/ml	Orange	Green
UID-39	morphine injection 10mg	Yellow	1ml ampoule registered as 15mg in 1ml ampoule
UID-569	ethinylestradiol + norethisterone tablet 35mcg + 1mg	Yellow	ethinylestradiol + norethisterone tablet 20mg + 10mg
UID-581	levonorgestrel-releasing plant two rod levonorgestrel-releasing implant each contains 75mg total 150mg	Yellow	levonorgestrel intrauterine system containing 52mg of levonorgestrel
UID-599	potassium iodide tablet 60mg	Yellow	potassium iodide tablet 130mg
UID-612	ergometrine injection 200mcg in 1ml ampoule	Yellow	ergometrine injection 500mcg
	**Third Validation (DRAP-** LIST-3b = 179 (See Fig 4a and 4b)	-	
	(93 yellow, 39 orange and 47 purple)		

LIST-03 containing 195 total unregistered medicines initially categorized as 37 (orange), 32 (purple) and 126 (yellow) was shared with two experienced pharmacists working in public health tertiary care hospital and at private retail pharmacy. Yellow color code was assigned to the remaining unregistered medicines. Out of the provisional 195 unregistered medicines, the status of 6 medicines was confirmed as registered and 16 medicines were changed from yellow to purple category to form LIST-3a. The LIST-3a containing 189 total unregistered medicines (104 yellow, 38 orange and 47 purple) was further confirmed for registration status by using MIS system available on the official website of DRAP. Out of these 189 unregistered medicines, a total of 16 medicines were confirmed as registered, including 10 from yellow and 4 from orange category (Table 2). Second and third validation resulted in shifting of 5 medicines from yellow to orange list to form LIST-3c which consisted of 179 total medicines (39 orange: LIST-4a, 47 purple: LIST-5a, and 93 yellow: LIST-6a) which was shared with DRAP for final confirmation of registration status (Table 2).

**Fig 4:**
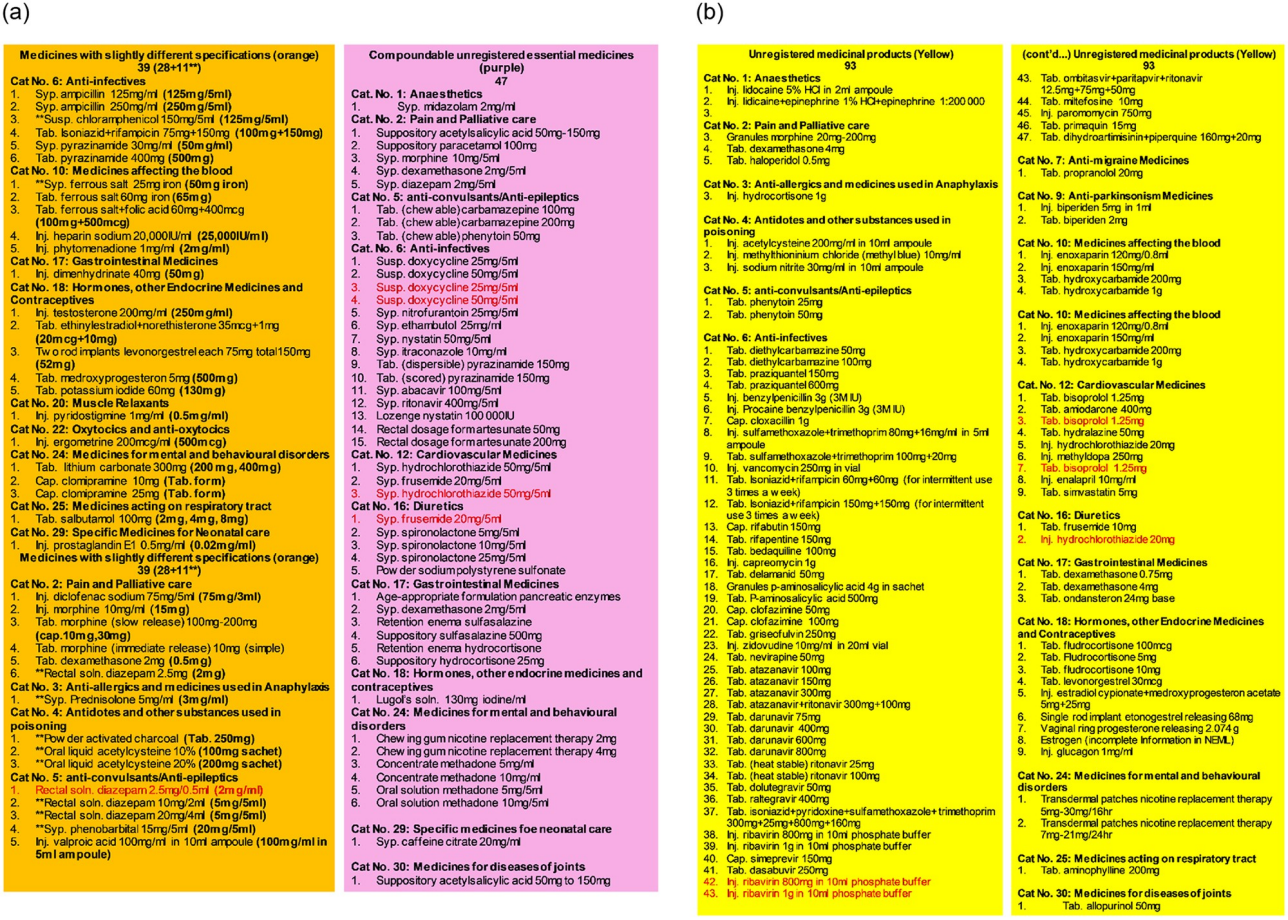
**a**. List and color codes of 179 unregistered medicines found in 19 categories of NEMLPK-2018 (List 4a (orange) and 5a (purple) medicines with slightly different specifications and compoundable unregistered essential medicines). **b**. List and color codes of 179 unregistered medicines found in 19 categories of NEMLPK-2018 (List 6a (yellow)—remaining unregistered essential medicines). *Red colored font indicates the medicines that appeared more than once in NEMLPK-2018. ** These medicines registered with slightly different specifications can also be prepared extemporaneously.

Cross validation of the data revealed 10 UIDs were identified to be duplicated in the data of 179 unregistered medicines which are highlighted with red colored font in the Fig 4a and 4b. The duplicated medicines were not corrected for their UID numbering to prevent ambiguity of data. Total 46 duplications were found in the 690 medicines as detailed in the S3 File.

### Step five: Finalization of lists

Five lists were generated after validation process including, LIST-02: List of registered Medicines (green, 591 medicines), List No. 3c: List of unregistered medicines (red, 179 medicines). The latter was further grouped into 3 lists; List 4a: List of medicines registered with slightly different specifications (Orange, 39 medicines), List 5a: List of compounding and age-appropriate formulations (purple, 47 medicines) and List No. 6a: List of remaining unregistered medicines not having any information about their registration status (Yellow, 93 medicines).

Fig 1 illustrates provides the overview of the process and generation of LIST-07, LIST-3c, LIST-4a, LIST-5a, and LIST-6a. The description of the medicines included in the latter 4 lists comprising of the medicines lacking registration status is shown in Fig 4a and 4b. Table 2 provides the details of the results of the validation process with details of medicines and reallocation of the color code. Summary of results is presented in Box 2.

**Box 2. Summary of results**

**List of total unregistered medicines (yellow + purple + orange)**

Out of 690 medicines, 179 (25.9%) medicines were found to be un-registered means no data was available about these medicines in information sources that were used to confirm the registration status of medicines

**List of unregistered medicines with no rectification (yellow)**

Out of these 93 (51.9%) medicines also did not have any closely related registered medicine or could be included in the compoundable items list and were kept in yellow list status.

**List of medicines with slightly different specifications (orange)**

Out of 179 unregistered items, 39 (21.78%) medicines were found with slightly different specifications in information sources that were used to confirm the registration status. 11 medicines among this category were identified as medicines that can be prepared using compounding.

**List of dispensing/compounding medicines (purple)**

Out of 179 unregistered items, 47 (26.2%) medicines were found as compounding and age-appropriate preparations.

**List of medicines with wrong or missing specifications**

See S4 File

**Category-wise %age of medicines lacking registration status**

See Table 3

**Fig 5:**
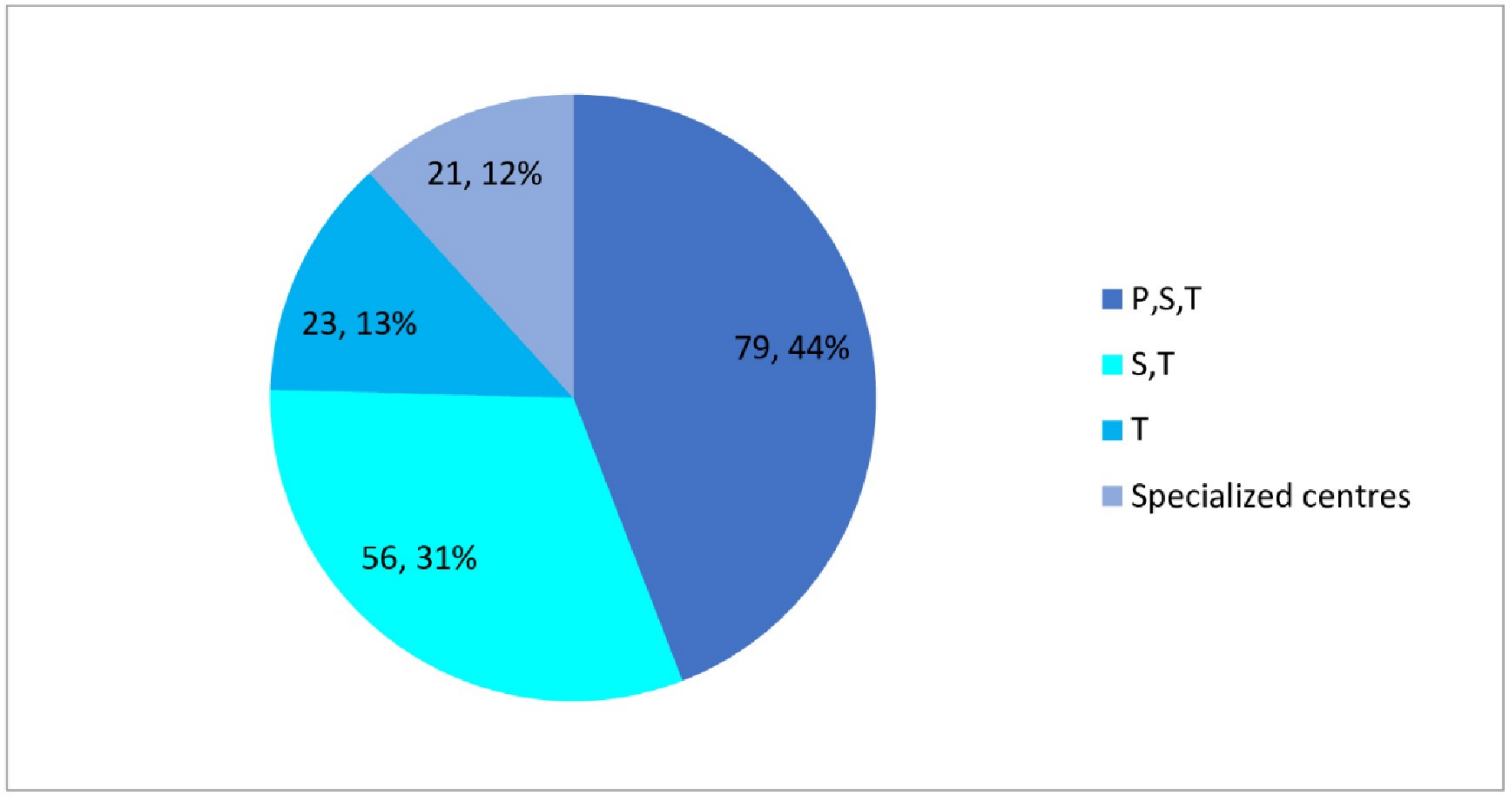
Distribution of unregistered essential medicines according to the health facility level.

**Fig 6:**
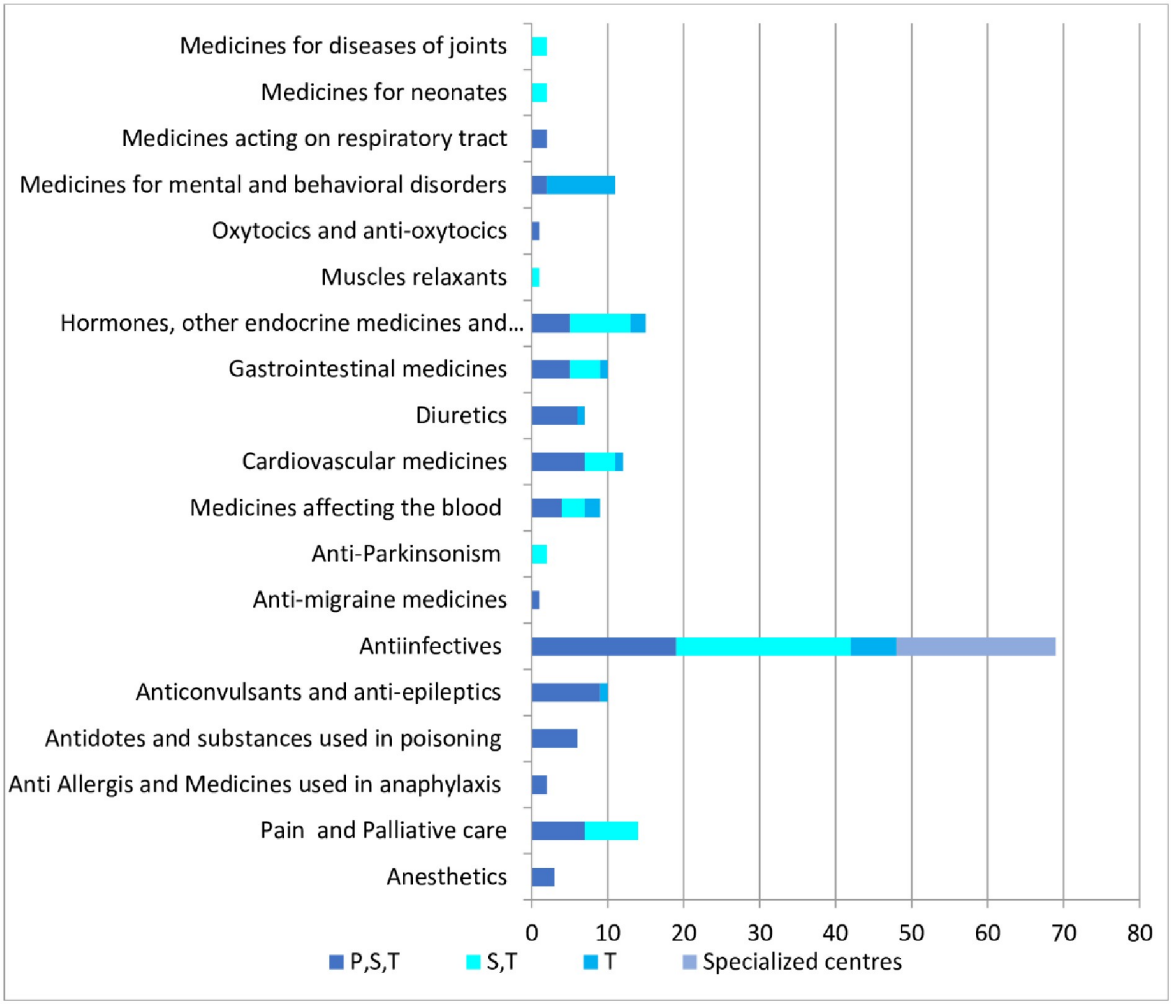
Category wise distribution of unregistered medicines at various facility levels.

The impact of un-registration status on each category was analysed by using the formula mentioned in methods and is enlisted in Table 3. The distribution of unregisterered medicines at the various health facility levels (primary, secondary and tertiary centres as well as specialized centres like for National TB control) are graphically depicted in Fig 5. The category wise distribution of the unregistered medicines with their healthy facility is shown in Fig 6.

**Table 3:** Descriptive summary for 690 medicines in NEML according to registration status.

No.*	Therapeutic Category	Generics	Expanded medicines	Registered medicines	Unregistered medicines			
					Slightly different specifications (O = orange)	Compounding & Age-Appropriate preparations (P = purple)	Remaining (Y = yellow)	Total (Y+O+P)
								Number, %ǂ
1	Anesthetics	13	23	20	00	01	02	03 (13%)
2	Medicines for pain and palliative care	19	53	39	05+01**	05	03	14 (26%)
3	Anti-allergics and medicines used in anaphylaxis	07	16	14	01**	00	01	02 (13%)
4	Anti-dotes and other substances used in poisoning	08	10	04	03**	00	03	06 (60%)
5	Anti-convulsants/Anti-epileptics	06	24	14	01+04**	03	02	10 (42%)
6	Anti-infective Medicines	111	254	185	05+01**	15	48	69 (27%)
7	Anti-migraine medicines	06	10	09	00	00	01	01 (10%)
9	Anti-parkinsonism medicines	01	02	00	00	00	02	02 (100%)
10	Medicines affecting the blood	16	36	27	04+1**	00	04	09 (25%)
12	Cardiovascular medicines	39	78	66	00	03	09	12 (15%)
16	Diuretics	06	15	08	00	05	02	07 (47%)
17	Gastro-intestinal medicines	18	41	31	01	06	03	10 (24%)
18	Hormones, other Endocrine medicines and contraceptives	30	40	25	05	01	09	15 (38%)
20	Muscle relaxants	06	09	08	01	00	00	01 (11%)
22	Oxytocics and Anti-oxytocics	04	06	05	01	00	00	01 (17%)
24	Medicines for mental and behavioral disorders	15	37	26	03	06	02	11 (30%)
25	Medicines acting on respiratory tract	07	20	18	01	00	01	02 (25%)
29	Specific medicines for neonatal care	06	08	06	01	01	00	02 (25%)
30	Medicines for diseases of joints	07	08	06	00	01	01	02 (25%)
	**Total**	**325**	**691**	**511**	28+ (11**)	**47**	**93**	**179 (26%)**

*Therapeutic Category Number according to NEMLPK-2018, 2018,

^ǂ^ = %age of medicines lacking proof of registration

**These medicines registered with slightly different specifications can also be prepared extemporaneously.

### List of medicines having incomplete information, wrong specifications and typographic mistakes in NEMLPK-2018

There were 15 medicines that were identified for having incomplete information for their strength, dosage form and pack size. Some medicines in the NEMLPK-2018 had wrong specifications or typographic mistakes like in the case of ampicillin suspension and salbutamol tablet. These 16 medicines were also checked in 20^th^ WHO model list of EMs 2017 [8] and the related information was summarized in S4 File.

## Discussion

This is a first study of its kind that analyses the NEML for important requisites regarding availability of medicines using comprehensive document analysis using sources other than NEMLPK. Publications related to availability of EMs in Pakistan are scarce and are limited to market surveys carried out on a few selected EMs mostly including registered medicines [25–28]. There are some studies based on qualitative data as well [15, 29]. None of these studies aimed to provide information on the complete range of EMs included in a particular category. Every year, number of papers are published on access to medicines world-wide but very few publications provide information on the registration status. A global access study carried out in 137 countries on antifungals showed the percentage of countries having license for injectable amphotericin B, oral and injectable itraconazole, and fluconazole to be 14.2%, 2.4%, 72.4%, and 0%, respectively. The percentage of countries with non-availability status was somewhat higher than for licensing figures except for fluconazole that was available in 5 countries under special programs [30]. The recent literature on availability of EMs in Pakistan is still scarce, sporadically covering only a few classes of medicines including cardiovascular [27, 28], anti-infectives [30, 31] respiratory medicines [25] along with some reports and studies on generalized group of medicines [13, 14, 26] None of the studies covered the complete range of EMs for the studied category but include only a few selected medicines.

### The process for selection of study methodology

This is a novel study that provides a concise methodology for comprehensive analysis of the NEML by going through the first step of documenting the registration status of the essential medicines. The study provides the information about the weightage and magnitude of this issue, which is generally overlooked in the literature regarding availability of medicines. In order to assess the availability of the EMs, the list of medicines described in the NEMLPK-2018, 7^th^ Edition issued by Drug Regulatory Authority of Pakistan was used [9]. The use of this list for the public availability through DRAP Mobile Application is the evidence of it being the last updated official version of NEMLPK. There are total 30 therapeutic categories in NEMLPK-2018, out of which, 19 were analyzed in this study for checking of registration status of medicines. The 19 categories selected for analysis mainly included those that were discussed previously in literature for shortage and non-availability. The current study generates two basic lists of EMs that constitute the list of medicines that are of ‘registered’ and ‘unregistered’ status in Pakistan. Both lists hold different strategies for further availability study. The measures for the maximizing their availability in the country would also differ. Only the medicines with registration status can technically be taken to clinical sites for on-spot physical availability surveys using well-defined and tested methodologies like WHO/HAI methodology [32]. According to Drug Act 1976 of Pakistan, “the production of every drug should be sufficient enough to meet the requirements of people and market supply of medicines should not be decreased”. It is also written in the act that “the manufacturer of medicines can-not stop the manufacturing of a period that that can result in medicines shortages” [33]. However, this law is applicable only for the medicines that are registered with DRAP. Many LMICs including Pakistan lack the notification system of medicines shortages. Systematic documentation of medicine shortages will better help in the analysis of strategies for prioritization and policy intervention [3].

### Medicines shortages, market authorization and impact on health system [6]

Market authorization is critical to medicines access and needs to be led by the disease burden data in order to suffice to the local needs of the population [34]. Medicine shortages adversely affect the health care system in number of ways [35], including potential of infiltration of substandard and falsified medicines in pharmaceutical supply chain [36, 37]. The current study shows 26% of essential medicines from 19 categories to be lacking the registration status and hence being prone to be smuggled into the country illegally or increase in prevalence of falsified medicines [38–40]. Reports of seizures of medicines imported illegally are also brought to media time to time [39, 41–43].

### Comparison with current evidence

A quarter (26%) of the EMs enlisted in the 19 studied categories of NEMLPK-2018 were found not registered in Pakistan, and hence do not possess the license for sales and distribution in the country. WHO/HAI criteria for grading fairly-high availability calls for the countries to ensure a minimum of 80% availability of EMs with the availability defined with respect to the percentage of surveyed facilities where the studied medicine was found available [6]. So far, there is no criteria to show the impact of absence of large number of medicines from a particular category of NEML.

The results of the study shows that none of the studied categories possessed 100% registration of the enlisted EMs (Table 3). The percentage of un-registered medicines ranged from 10–100%. The categories showing high proportion of unregistered medicines included anti-Parkinson’s medicines (100%), antidotes and other substances used in poisoning (60%), diuretics (47%), anticonvulsants/antiepileptics (42%), hormones and other endocrine medicines and contraceptives (38%), medicines for mental and behavioral disorders (30%), anti-infectives (27%), medicines for pain and palliative care (26%), specific medicines for neonatal care (25%), medicines for diseases of joint (25%), gastrointestinal medicines (24%) and cardiovascular drugs (15%) (Table 3). WHO analysis of medicine shortages with respect to global approaches for addressing the shortages of EMs in health systems indicates antibiotics, anti-cancers, cardiovascular medicines and anesthetics as the key therapeutic classes facing medicine shortages globally [3].

This study presents a new parameter in understanding the magnitude of the problem by calculating the category-wise %age of medicines lacking registration status. The analysis of identified unregistered medicines also shows that almost half of the identified unregistered EMs can have improved availability by strengthening compounding services, prioritizing registration of age-appropriate preparations, and developing a policy framework for revising the NEML or the specifications of preparations already registered with slightly different specifications.

In a 2018 survey conducted on 26 selected Access group antibiotics the mean availability was found to be less than 45%. The survey recorded complete non availability of cefazolin, chloramphenicol, nitrofurantoin, cloxacillin, phenoxymethylpenicillin, spectinomycin in community private retail pharmacies [31]. The current study confirms the registration status of the later 6 generics as 13 EMs, except for cloxacillin 1g capsules.

A study conducted in 2013 showed absolute non availability of beclomethasone, budesonide in any of the public or private survey sites as well as in national procurement center in Pakistan [25]. Despite, the fact that beclomethasone inhaler was registered in Pakistan in 2013.

Among the older reports, study by Rasheed *et al*.in 2005 on 56 essential and clinically important medicines showed that 49 medicines to be either short or not available in the market with 29 medicines (59%) lacking registration status. The missing EMs included cardiovascular drugs (22.4%), poisoning antidotes (16.3%), and anticonvulsants (10.2%) [14]. The 2001 list of missing EMs by Network for Consumer Protection included cyclizine 50 mg injection and tablets, amitryptyline 10 mg, 25 mg and 50 mg tablets, sodium valproate 200 mg tablet and 200 mg syrup, baclofen 10 mg tablets, spironolactone 25 mg tablets, glyceryl trinitrate 0.5 mg tablets, thyroxin 50 mcg tablets, digoxin 250 mcg, griseofulvin 125 mg tablets, benzathine penicillin injection, azathioprine 50 mg tablets. However, these reports mainly targeted the absence of the popular brands from the market. Only spironolactone and baclofen tablets were reported to have no alternates/generic substitutes at that time.

### Antidotes and medicines used in poisoning management

The antidotes sodium nitrite and activated charcoal along with dimercaprol, ipecacuanha syrup, pralidoxime mesylate, penicillamine and protamine injection were found not available in the study carried out in 2005 with only the latter three medicines as unregistered medicines [14]. In the current study 4 medicines were found unregistered including activated charcoal powder, acetylcysteine injection and oral liquid, methylthioninium chloride (methyl blue), and sodium nitrite injection. Activated charcoal is enlisted in Pharmaguide [19] as 250 mg capsules. Activated charcoal is also called universal antidote [44] and is considered a household aid [45] to reduce absorption and active elimination of poisons [46]. It is available internationally in variety of forms including granules 50 g packing as well as oral suspension 200 mg/mL [47] and can also be prepared using compounding techniques [48]. Injectable acetylcysteine 200 mg/mL is used for the treatment of acetaminophen toxicity [49, 50], it is available in Pakistan only as sachets for nebulization. Methylthioninium chloride and sodium nitrite are used as an antidote to treat methaemoglobinaemia [51] and cyanide intoxication, respectively. The latter is used in combination with sodium thiosulfate [52] as listed in WHO model EML.

### Medicines for central nervous system disorders

Among the shortages reported for medicines for mental and behavioral disorders haloperidol injection is quoted in the study from 2005 [14]. Earlier studies from Pakistan have also reported the shortage and non-availability of anticonvulsants including phenytoin injection and syrup, phenobarbitone tablets, elixir, and injection [13, 14, 29] The qualitative study conducted by Atif M *et al*. showed that the tranquilizers and other medicines acting on CNS were short in supply [29]. Apart from this phenobarbitone injectable 200 mg/2mL and oral liquid 20 mg/5mL varied in specifications from those cited in EML of Pakistan as 200 mg/mL and oral liquid were 15 mg/5mL, respectively. Sodium valproate preparation is registered as 500 mg/5mL ampoule instead of the 1000 mg/10mL ampoule enlisted in NEMLPK-2018. Similarly, for diazepam rectal solutions the three strengths mentioned in the NEMLPK-2018 differed from those found as registered medicines (Fig 4a and 4b). In total, 21 medicines were found unregistered in these two categories (S2 File)

### Anti-infectives shortage and missing drugs in Pakistan

Anti-infectives constitute the largest category of NEML containing total 104 generics comprising of 254 medicines. Out of these, 69 medicines were found unregistered. Anti-infectives were further divided into various sub-categories. In sub-category of anthelmintics diethylcarbamazine tablets 50 mg and 100 mg and praziquantel tablet 150 mg and 600 mg were unregistered. The antibacterials sub-category contained 136 medicines (55 total generics), out of which 25 medicines were found unregistered including 12 first line WHO Access group antibacterials including benzathine penicillin 3 MIU injection, procaine penicillin 3MU injection, Chloramphenicol 1 g Injection Oil based, cloxacillin 1 g injection and vancomycin 250 mg injection (S2 File). Benzathine penicillin is a classic example of medicines facing chronic short supply at global level [3]. Out of the 53 anti-virals, 24 were found unregistered including the entire class of protease inhibitors. The results of study conducted by Atif et al. also showed that many anti-virals like acyclovir were short in supply [29].

Non-availability of some essential anti-infectives have become a global issue. Amphotericin B injection was reported as unregistered till 2005 in Pakistan [14]. A global study reports this important antifungal to be unregistered in 22 out of 144 countries [30]. Despite the current registration status of Amphotericin B colloidal preparation, the availability is questionable and would need documentation through physical availability survey.

Medicines access for neglected tropical diseases including malaria, tuberculosis, and leishmaniasis also face persistent barriers. Many anti-parasitics drugs, like miltefosine and parmomycin among anti-leishmaniasis drugs and artesunate rectal dosage form 50 mg and 200 mg and primaquine 15 mg tablets among anti-malarials were found unregistered from NEMLPK-2018. Anti-TB medicines p-amino salicylic acid and clofazimine preparations as well as single ingredient anti-TB preparations in age-appropriate form as well as few combinations were also not registered despite inclusion in the National TB program. Lack of registration, difficulty in import and non-availability of quality assured antileishmanial medicines have been reported for Pakistan in 2016 [53]. In 2018 almost 21,000 people suffered from Leishmaniasis in KPK province of Pakistan [54] and the medicine were acquired through special import permission but delay in custom clearance of glucantime (meglumine antimoniate) injection worsened the situation [54]. In 2017, the outbreak in Africa caused the global shortage of glucantime that also affected the supply of glucantime to Pakistan by Medicines Sans Francis (MSF) which shows that the supply of medicines is a global problem and cannot be dealt in isolation [55]. Sonyoto *et al*. analysed the causes of post-registration access failure of miltefosine, another related drug licensed for the treatment of visceral leishmaniasis [56].

### Cardiovascular drugs

Total 78 medicines (39 generics) were present in therapeutic category of cardiovascular medicines, out of which 14 were found unregistered (S2 File). Medicines used in heart failure bisoprolol tablet 1.25 mg, enalapril tablet 2.5 mg, enalapril injection 10 mg/mL, frusemide oral liquid 20 mg/5mL, hydrochlorothiazide 50 mg/5mL were found unregistered. Lipid lowering agent simvastatin tablet 5 mg was also found unregistered. Hydralazine injection, methyldopa and spironolactone were either short in supply or unregistered [14]. Another study by Atif et. al. carried out in 2019 also showed that cardiovascular and anti-hypertensive drugs were short in supply in Pakistan [29]. A comparison done in 2010 showed the poor availability (26–57%) of captopril, losartan, nifedipine, atenolol and hydrochlorothiazide in public sector settings in Pakistan [57]. Another national survey on availability of 18 selected cardiovascular medicines was carried out in 2019. All the medicines included in the survey were registered in Pakistan and still showed fairly low availability especially in public sector [58].

### Compounding preparations

Current study reveals that out of the 180 unregistered medicines 47(26.11%) medicines have either the potential to be prepared through compounding services or are age-appropriate preparations. These include many cardiovascular and diuretics, pain and palliative care, gastrointestinal, mental, and behavioral disorders and anti-infectives preparations for geriatrics and pediatric use (Table 2). Most of these preparations included oral liquids and rectal preparation of solutions and suppositories that are prepared as extemporaneous or compounding preparations. Examples include activated charcoal powder [48], methadone oral solutions, griseofulvin preparations [59], itraconazole oral solution, [60, 61], diazepam rectal solution [62], Lugol’s solution, and glycerin suppositories. Compounding services hold key importance in access to age-appropriate preparations for children in particular and for availability of orphan medicines [48]. Pakistan has consistently reported to be of insufficient and not at par with international standards of practices in general pharmacy services [63]. Moreover, the regulations in Pakistan are very rudimentary regarding compounding services which warrants immediate steps to cover the gaps and improve access to EMs through supporting compounding services.

### Other age-appropriate preparations

Paediatric medicines often face non-availability and shortages issues [3]. Chewable tablets are palatable, stable, precise in dosing, ease in portability, and delivery. These are preferred for children and geriatrics due to lower risk of aspiration and are considered as age-appropriate preparations [64]. The phenytoin 50 mg, carbamazepine 100 mg and 200 mg, and albendazole 400 mg chewable tablets were not found registered in Pakistan despite that they have been listed as EMs in Pakistan. Phenytoin and carbamazepine are anti-epileptics drugs (AED’s) and are narrow therapeutic index drugs warranting dose accuracy and compliance [65]. Similarly, WHO model enlists 10 dispersible tablets in the anti-infective category whereas NEMLPK-2018 enlists only pyrazinamide 150 mg and artemether and lumefantrine 20+120 mg tablets both are not registered as dispersible tablets in Pakistan.

### Revision of NEML to resolve the inclusion of medicines with slightly different specifications

It was identified in the study of NEML that out of 179 of total unregistered medicines, 39 (21%) medicines were registered with slightly different specifications as detailed in Fig 4a (Orange text boxes). Hence either, the NEML should be revised to replace the medicines with the available specification in case no serious compromise in patient care is anticipated or the other way round, DRAP should proceed to ensure availability of medicines as per NEML specifications if defended by a solid scientific rationale and guide industry to come up with revised specification in a defined time frame.

### Multiple concentrations of injectable preparations

Enlisting of multiple concentration of injectable drugs in NEML is an area of safety concern For example, gentamicin injection is enlisted in two concentrations as 10 mg/mL and 20 mg/mL in NEML. In poor resource health system, availability of multiple concentrations of same injectable preparation in publicly accessible supplies can lead to misadventures like the one reported in 2019, when a concentrated injection of potassium chloride was inadvertently used in place of normal saline ampoule [66]. In case of registration of two concentration of a medicine especially for parenteral therapy, a strict control on circulation and training of health care professionals needs to be practiced.

### Health facility levels and impact of unregistered medicines

The results in Figs 5 and 6 show that 44% of the unregistered medicines are meant for utilization at all the three levels of health facility and hence must include essential medicines of crucial importance. Absence of these medicines from the regular pharmaceutical supply chain is clearly a hurdle to access to essential medicines across the country and needs immediate resolution.

### Implications on policy and practice

Prioritization mechanism should be defined for market authorization of EMs lacking registration status identified in this study which should be based on the current availability profile of therapeutic alternates. Emphasis should be on medicines that do not have suitable therapeutic alternates or are medicines for critical care and emergency use in life threatening conditions. Registration of EMs should also be ensured to control the prevalence of “imported” and falsified medicines in the country. The spread of “imported” medicines in the local pharmaceutical supply chain compromise the assurance of safety, and quality of EMs. Collaboration with international organizations working on access to essential and orphan medicines facing global shortages would also help the country in preparedness and timely action.

DRAP should develop a dedicated database management system and regulatory guidance on the reporting and developing systematic approach to deal with anticipated as well as actual medicine shortages faced by the country. Revision of NEML should be a continuous process and guided by evidence including the information obtained through this study on the registration status of EMs. Evidence based alternate modalities should be included for improving the patients access to treatment. Update of NEML should be made to include the medicines with slightly different specification registered in the country without compromising the rationale of their inclusion in the NEML list. In the latter case, the shift to the more rational specifications must be brought through the help of DRAP within a designated time-line.

The study identified compounding as an essential component of pharmacy services to help in the delivery of wide range of EMs especially including medicines for children. Measures should be taken for strengthening these services in Pakistan. Multi-sectorial consortium for availability of EMs in Pakistan should be formulated in order, to acquaint the stakeholders including regulators, industry, prescribers, pharmacy practitioners, and researchers with the gaps identified in access to EMs. The consortium should carry out activities and lead research work for awareness and collaboration of stakeholders on the unmet pharmaceutical needs of the country and especially EMs. Advocacy and awareness on the age-appropriate formulations must also be developed to promote the interest of manufacturers to ensure the availability of the EMs for all age groups.

Implementation of EMs lists guided by standard treatment guidelines is an extensive and long-term process with the two concepts invariably supporting each other. Mechanisms such as health insurance are likely to support these concepts. The insufficient coverage of medicines for Parkinson’s disease and antidotes and inclusion of obsolete therapeutics modalities like salbutamol oral dosage forms for asthma in NEMLPK-2018 shows that much needs to be done on the training of the health care professionals in this regard.

Provision of accessible and transparent information on the registration status of the medicine’s guards against the infiltration of falsified medicines in the pharmaceutical supply chain and is demanded for transparency in pharmaceutical supply and governance for ensuring universal health coverage. Public access to updated and reliable information on registered medicines should be improved in Pakistan. The MIS database introduced by DRAP in 2018 is still under development and does not provide the complete list of registered medicines. However, recent digitization of the authority through Pakistan Integrated Regulatory Management System (PIRIMS) with the support of United Stated States Pharmacopoeia Commission- Promoting Quality of Medicines Program (USP-PQM) is a promising step in this regard and should be strengthened.

### Limitations of the study

The study is based on publicly available documents regarding the registration status of the essential medicines. However, absence of reliable up-to-date information from regulatory sources in this respect poses key limitations to this study. Information collected from the private sources may lack timely updating in case of cancellation of registration or discontinuation of the product by the manufacturer. Hence, registration status of essential medicines provided in the information sources suggest that the product must have been registered at some time with the regulatory agency in Pakistan, however this study is unable to confirm the product’s current registration status.

To overcome this limitation, confirmation of the registration status was made through a process of validation using the limited information provided by DRAP on its webpage. However, the names of those essential medicines, who have no evidence of registration were shared with DRAP for confirmation.

It is clear, that the absence of registration status is a major barrier in the availability of medicines but at the same time the registration of medicine is not the confirmation of its market availability. The later can only be confirmed through a field survey.

## Conclusion

Although the availability of medicines can only be confirmed through a detailed physical availability survey, absence of market authorization marks the major barrier in ensuring the access to EMs in the country. The study results emphasized that the review of the market authorization should be carried out as a first step for proceeding on the assessment of non-availability of essential medicines in any country. Further research needs to be done for development of remedial strategies on improving access to EMs after a fact-finding study on the other reasons for such shortages. This study also calls for an inclusive approach to consult with the stakeholders and to develop strategies to ensure availability of EMs enlisted in the NEML of Pakistan.

## Supporting information

S1 File Form for extraction of data collection from information sources. (PDF)

S2 File Category wise expanded list of essential medicines with color-coding and result summary. (PDF)

S3 File List of medicines with multiple appearance in NEMLPK-2018. (PDF)

S4 File List of incomplete information, missing strength, wrong specifications, and typographic mistakes. (PDF)
