# Supplementary material for: Availability of essential medicines in Pakistan—A comprehensive document analysis
Source: PLoS One. 2021 Jul 9;16(7):e0253880. doi: 10.1371/journal.pone.0253880 (PMC8270130; doi:10.1371/journal.pone.0253880)
Supplement: S1 File — (PDF) [file pone.0253880.s001.pdf]

S1: Form for Extraction of Data Collection from information Sources

S1 File: CATEGORY NUMBER AND NAME OF THERAPEUTIC CATEGORY ACCORDING TO NEMLPK-2018

			NEMK-PK 2018			INFORMATION SOURCES									
						PHARMAGUIDE			PHARMAPEDIA PAKISTAN			DRUG INFO SYS			Remarks (if any)
**REGISTRATION STATUS**	**SECTOR (P/S/T)**	**UID**	**MEDICINE**	**DOSAGE FORM**	**STRENGTH**	**MEDICINE**	**DOSAGE FORM**	**STRENGTH**	**MEDICINE**	**DOSAGE FORM**	**STRENGTH**	**MEDICINE**	**DOSAGE FORM**	**STRENGTH**	
see legend below															

**SUMMARY**	**CATEGORY NUMBER AND NAME OF THERAPEUTIC CATEGORY ACCORDING TO NEMLPK-2018**				**Colors**	**Legends**	Abbreviation
**Generics**	**Expanded medicinal products**	**Unregistered medicinal products (Yellow)**	**Medicinal products with slightly different specifications (Orange)**	**Unregistered medicines can be available through compounding**		Registered	G
						Registered but with slightly different specifications	O
						Compounding + age-appropriate formulations	P
						Unregistered Medicinal products	Y
