# Supplementary material for: Availability of essential medicines in Pakistan—A comprehensive document analysis
Source: PLoS One. 2021 Jul 9;16(7):e0253880. doi: 10.1371/journal.pone.0253880 (PMC8270130; doi:10.1371/journal.pone.0253880)
Supplement: S2 File — (PDF) [file pone.0253880.s002.pdf]

S2 File: Category wise List of essential medicines with color-coding and result summary

NEML-2018				
1. ANESTHETICS				
SECTOR	UID	MEDICINE	DOSAGE FORM	STRENGTH
	GENERAL ANESTHETICS AND OXYGEN			
S,T	1	isoflurane	inhalational	
T	2	sevoflurane	inhalational	
P,S,T	3	nitrous oxide	inhalational	Medical gases supplied to hospital**
P,S,T	4	oxygen	inhalational (medicinal gas)	Medical gases supplied to hospital**
P,S,T	5	ketamine	injection	50mg/ml in 10ml vial
P,S,T	6	propofol	injection	10mg/ml
P,S,T	7	propofol	injection	20mg/ml
S, T	8	glycopyrrolate	injection	0.2mg/ml
	LOCAL ANESTHETICS			
S, T	9	Bupivacaine	injection	0.25% as hydrochloride
S, T	10	Bupivacaine	injection	0.5% as hydrochloride
P, S, T	11	lidocaine	injection	1% hydrochloride
P, S, T	12	lidocaine	injection	2% hydrochloride
P, S, T	13	lidocaine	injection	5% hydrochloride in 2ml ampule
P, S, T	14	lidocaine	gel	2%w/v 15gm tube
P, S, T	15	lidocaine+epinephrine (adrenaline)	dental cartridge	2% hydrochloride+epinephrine 1:80 000
P, S, T	16	lidocaine+epinephrine (adrenaline)	injection	1% hydrochloride or sulphate+epinephrine 1:200 000
P, S, T	17	lidocaine+epinephrine (adrenaline)	injection	2% hydrochloride or sulphate+epinephrine 1:200 000
S, T	18	ephedrine	injection	30mg in 1ml vial
		PREOPERATIVE MEDICATION AND SEDETION FOR SHORT TERM PROCEDURE		
P, S, T	19	atropine	injection	1mg in 1 ml ampule
P, S, T	20	midazolam	injection	1mg/ml
P, S, T	21	midazolam	oral liquid	2mg/ml
P, S, T	22	midazolam	tablet	7.5mg
P, S, T	23	midazolam	tablet	15mg
**Generics**	**Expanded medicinal products**	**Unregistered medicinal products (Yellow)**	**Medicinal products with slightly different specifications (Orange)**	**Unregistered medicines can be available through compounding**
**13**	**23**	**2**	**0**	**1**

S2: Category wise List of essential medicines with color-coding and result summary

NEML-2018				
**2.MEDICINES FOR PAIN AND PALLIATIVE CARE**				
**SECTOR**	**UID**	**MEDICINE**	**DOSAGE FORM**	**STRENGTH**
**NON-OPIOID ANALGESICS, ANTI-PYRETICS AND NON-STEROIDAL ANTI INFLAMMATORY DRUGS**				
P, S, T	24	acetylsalicylic acid	suppository	50mg-150mg
P, S, T	25	acetylsalicylic acid (aspirin)	tablet	75mg EC
P, S, T	26	ibuprofen	suspension	100mg/5ml
P, S, T	27	ibuprofen	tablet	400mg
P, S, T	28	paracetamol	syrup	120mg/5ml
P, S, T	29	paracetamol	suppository	100mg
P, S, T	30	paracetamol	injection	150mg/ml
P, S, T	31	paracetamol	tablet	500mg
S, T	32	diclofenic sodium	tablet	50mg
S, T	33	diclofenic sodium	injection	75mg/5ml * 75mg/3ml
S, T	34	naproxen	tablet	250mg
S, T	35	naproxen	tablet	500mg
**OPIOID ANALGESICS**				
T	36	codeine	tablet	30mg
T	37	nalbuphine HCl	injection	10mg/ml
S, T	38	morphine	granules	20mg-200mg
S, T	39	morphine	injection	10mg in 1 ml ampule *15mg/ml
S, T	40	morphine	oral liquid	10mg/5ml
S, T	41	morphine	tablet (slow release)	10mg-200mg * caps 10mg, 30mg
S, T	42	morphine	tablet (immediate release)	10mg * simple tablet
**MEDICINES FOR OTHER COMMON SYMPTOMS IN PALLIATIVE CARE**				
T	43	amitriptyline	tablet	10mg
T	44	amitriptyline	tablet	25mg
P, S, T	45	cyclizine	injection	50mg/ml
P, S, T	46	cyclizine	tablet	50mg
P, S, T	47	dexamethasone	injection	4mg/ml in 1ml ampule
P, S, T	48	dexamethasone	oral liquid	2mg/5ml
P, S, T	49	dexamethasone	tablet	2mg * 0.5mg
P, S, T	50	dexamethasone	tablet	4mg
P, S, T	51	diazepam	injection	5mg/ml
P, S, T	52	diazepam	oral liquid	2mg/5ml
P, S, T	53**	diazepam	rectal solution	2.5mg * 2mg
P, S, T	54	diazepam	rectal solution	5mg
P, S, T	55	diazepam	rectal solution	10mg
P, S, T	56	diazepam	tablet	5mg

P, S, T	57	diazepam	tablet	10mg
S, T	58	haloperidol	injection	5mg in 1ml ampule
S, T	59	haloperidol	oral liquid	2mg/ml
S, T	60	haloperidol	solid oral dosage form	0.5mg
S, T	61	haloperidol	solid oral dosage form	2mg
S, T	62	haloperidol	solid oral dosage form	5mg
P, S, T	63	loperamide	solid oral dosage form	2mg
P, S, T	64	metoclopramide	injection	5mg/ml in 2ml ampule
P, S, T	65	metoclopramide	oral liquid	5mg/5ml
P, S, T	66	metoclopramide	solid oral dosage form	10mg as HCL
S, T	67	ondansteron	injection	2mg base/ml in 2ml ampule
S, T	68	ondansteron	oral liquid	4mg base/5ml
S, T	69	ondansteron	solid oral dosage form	equivalent to 4mg base
S, T	70	ondansteron	solid oral dosage form	equivalent to 8mg base
P, S, T	71	bisacodyl	tablet	5mg
S, T	72	pregabalin	capsules	75mg
S, T	73	pregabalin	capsules	150mg
S, T	74	pregabalin	capsules	300mg
S, T	75	tramadol	capsules	50mg
S, T	76	tramadol	injection	50mg/ml
**Generics**	**Expanded medicinal products**	**Unregistered medicinal products (Yellow)**	**Medicinal products with slightly different specifications (Orange)**	**Unregistered medicines can be available through compounding**
**19**	**53**	**3**	**6**	**5**

S2: Category wise List of essential medicines with color-coding and result summary

NEML-2018				
**3. ANTI-ALLERGICS AND MEDICINES USED IN ANAPHYLAXIS**				
**SECTOR**	**UID**	**MEDICINE**	**DOSAGE FORM**	**STRENGTH**
**P, S, T**	77	chlorpheniramine	tablets	4mg
**P, S, T**	78	chlorpheniramine	injection	10mg/ml in 1ml ampule
**P, S, T**	79	dexamethasone	injection	4mg/ml in 1ml ampule
**P, S, T**	80	epinephrine (adrenaline)	injection	1mg/1ml ampule
**P, S, T**	81	hydrocortisone	powder for injection	100mg
**P, S, T**	82	hydrocortisone	powder for injection	250mg
**P, S, T**	83	hydrocortisone	powder for injection	1gm
**P, S, T**	84	loratadine	oral liquid	1mg/ml
**P, S, T**	85	loratadine	tablets	10mg
**P, S, T**	86	prednisolone**	oral liquid	5mg/ml * 3mg/ml
**P, S, T**	87	prednisolone	tablets	5mg
**P, S, T**	88	prednisolone	tablets	25mg
**P, S, T**	89	pheniramine (maleate)	injection	22.7mg/ml in 2ml ampule
**P, S, T**	90	pheniramine (maleate)	tablets	25mg
**P, S, T**	91	pheniramine (maleate)	tablets	50mg
**P, S, T**	92	pheniramine (maleate)	elixir/syrup	15mg/5ml
**Generics**	**Expanded medicinal products**	**Unregistered medicinal products (Yellow)**	**Medicinal products with slightly different specifications (Orange)**	**Unregistered medicines can be available through compounding**
**7**	**16**	**1**	**1**	**0**

S2: Category wise List of essential medicines with color-coding and result summary

NEML-2018				
4. ANTI-DOTES AND OTHER SUBSTANCES USED IN POISONING				
**SECTOR**	**UID**	**Medicine**	**Dosage form**	**Strength**
**NON-SPECIFIC**				
P, S, T	93	activated charcoal**	powder	* tablet 250mg
**SPECIFIC**				
P, S, T	94	acetylcysteine	injection	200mg/ml in 10ml ampule
P, S, T	95	acetylcysteine**	oral liquid	10 percent (10%) * 100mg sachets
P, S, T	96	acetylcysteine**	oral liquid	20 percent (20%) * 200mg sachets
P, S, T	97	atropine	injection	1mg (Sulphate) in 1ml ampule
P, S, T	98	calcium gluconate	injection	100 mg/ml in 10ml ampule
P, S, T	99	methylthioninium chloride (methyl blue)	injection	10mg/ml in 10ml ampule
P, S, T	100	naloxone	injection	400 micrograms in 1ml ampule
P, S, T	101	sodium nitrite	injection	30mg/ml in 10ml ampule
P, S, T	102	deferoxamine	powder for injection	500mg in vial
**Generics**	**Expanded medicinal products**	**Unregistered medicinal products (Yellow)**	**Medicinal products with slightly different specifications (Orange)**	**Unregistered medicines can be available through compounding**
**8**	**10**	**3**	**3**	**0**

S2: Category wise List of essential medicines with color-coding and result summary

NEML-2018				
5. ANTI-CONVULSANTS/ANTI-EPILEPTICS				
**SECTOR**	**UID**	**MEDICINE**	**DOSAGE FORM**	**STRENGTH**
P, S, T	103	carbamazepine	oral liquid	100mg/5ml
P, S, T	104	carbamazepine	tablet (chewable)	100mg
P, S, T	105	carbamazepine	tablet (chewable)	200mg
P, S, T	106	carbamazepine	tablet (scored)	100mg
P, S, T	107	carbamazepine	tablet (scored)	200mg
P, S, T	108	diazepam**	gel or rectal solutions	5mg/ml in 0.5ml tubes =2.5mg/0.5ml *2mg/ml
P, S, T	109	diazepam**	gel or rectal solutions	5mg/ml in 2ml tubes =10mg/2ml * 5mg/5ml
P, S, T	110	diazepam**	gel or rectal solutions	5mg/ml in 4ml tubes =20mg/4ml * 5mg/5ml
P, S, T	111	magnesium sulfate	injection	0.5g/ml in 2ml ampoule (equivalent to 1 gram in 2ml)
P, S, T	112	magnesium sulfate	injection	0.5g/ml in 10ml ampoule (equivalent to 5grams in 10ml)
P, S, T	113	phenobarbital	injection	200mg/ml
P, S, T	114	phenobarbital**	oral liquid	15mg/5ml *20mg/5ml
P, S, T	115	phenobarbital	tablet	15mg to 100mg
P, S, T	116	phenytoin	injection	50mg/ml in 5ml vial
P, S, T	117	phenytoin	oral liquid	25-30mg/5ml
P, S, T	118	phenytoin	solid oral dosage form	25mg
P, S, T	119	phenytoin	solid oral dosage form	50mg
P, S, T	120	phenytoin	solid oral dosage form	100mg
P, S, T	121	phenytoin	tablet (chewable)	50mg
P, S, T	122	valproic acid (sodium valproate)	oral liquid	200mg/5ml
P, S, T	123	valproic acid (sodium valproate)	tablet (enteric coated)	200mg
P, S, T	124	valproic acid (sodium valproate)	tablet (enteric coated)	500mg
T	125	valproic acid (sodium valproate)	injection	100mg/ml in 4ml ampoule
T	126	valproic acid (sodium valproate)	injection	100mg/ml in 10ml ampoule (1000mg) *100mg/ml in 5ml ampoule
**Generics**	**Expanded medicinal products**	**Unregistered medicinal products (Yellow)**	**Medicinal products with slightly different specifications (Orange)**	**Unregistered medicines can be available through compounding**
**6**	**24**	**2**	**5**	**3**

S2: Category wise List of essential medicines with color-coding and result summary

NEML-2018				
6. ANTI-INFECTIVES				
**SECTOR**	**UID**	**MEDICINE**	**DOSAGE FORM**	**STRENGTH**
**6.1 ANTHELMENTICS**				
**6.1.1 INTESTINAL ANTHELMENTICS**				
P, S, T	127	albendazole (chewable)	tablet	400mg
P, S, T	128	albendazole	oral liquid	100mg/5ml
P, S, T	129	mebendazole	tablet	100mg
P, S, T	130	mebendazole	tablet	500mg
P, S, T	131	pyrental	oral liquid	50mg/ml
P, S, T	132	pyrental	tablet (chewable)	250mg
T	133	ivermectin	tablet	3mg
T	134	levamisole	tablet	50mg
T	135	levamisole	tablet	150mg
**6.1.2 ANTI-FILARIALS**				
P, S, T	136	diethylcarbamazine	tablet	50mg
P, S, T	137	diethylcarbamazine	tablet	100mg
**ANTISCHISTOSOMALS AND OTHER ANTI-TREMATODE MEDICINES**				
S, T	138	praziquantel	tablet	150mg
S, T	139	praziquantel	tablet	600mg
**6.2 ANTI-BACTERIALS**				
**BETA-LACTAM ANTI-BIOTICS**				
**KEY ACCESS ANTI-BIOTICS**				
P, S, T	140	amoxicillin	powder for oral liquid	125mg/5ml
P, S, T	141	amoxicillin	powder for oral liquid	250mg/5ml
P, S, T	142	amoxicillin	injection	250mg
P, S, T	143	amoxicillin	injection	500mg
P, S, T	144	amoxicillin	solid oral dosage form	250mg
P, S, T	145	amoxicillin	solid oral dosage form	500mg
S, T	146	amoxicillin + clavulanic acid	oral liquid	125mg amoxicillin + 31.25mg clavulanic acid
S, T	147	amoxicillin + clavulanic acid	oral liquid	250 mg amoxicillin + 62.5mg clavulanic acid
S, T	148	amoxicillin + clavulanic acid	tablet	500mg amoxicillin + 125mg clavulanic acid

P, S, T	149	ampicillin	powder for injection	500mg
P, S, T	150	ampicillin	powder for injection	1gm
P, S, T	151	ampicillin	capsules	250mg
P, S, T	152	ampicillin	capsules	500mg
P, S, T	153	ampicillin	syrups	125mg/ml *125mg/5ml
P, S, T	154	ampicillin	syrups	250mg/ml *250mg/5ml
P, S, T	155	benzathine benzylpenicillin	powder for injection	900mg benzylpenicillin (1.2 million IU) in 5ml vial
P, S, T	156	benzathine benzylpenicillin	powder for injection	1.44g benzylpenicillin (2.4 million IU) in 5ml vial
P, S, T	157	benzylpenicillin	powder for injection	600mg (1 million IU) in vial
P, S, T	158	benzylpenicillin	powder for injection	3grams (3 million IU) in vial
P, S, T	159	phenoxymethylpenicillin	powder for oral liquid	250mg/5ml
P, S, T	160	phenoxymethylpenicillin	tablet	250mg
P, S, T	161	procaine benzyl penicillin	powder for injection	1 gram (1 million IU) In vial
P, S, T	162	procaine benzyl penicillin	powder for injection	3 grams (3 million IU) in vial
P, S, T	163	cefalexin	powder for reconstitution with water	125mg/5ml
P, S, T	164	cefalexin	powder for reconstitution with water	250mg/5ml
P, S, T	165	cefalexin	solid oral dosage form	250mg
P, S, T	166	cefazolin	powder for injection	1gm in vial
P, S, T	167	cloxacillin	capsules	500mg
P, S, T	168	cloxacillin	capsules	1gram
P, S, T	169	cloxacillin	powder for injection	500mg in vial
P, S, T	170	cloxacillin	powder for oral liquid	125mg/5ml
S, T	171	cloxacillin + ampicillin	powder for suspension	125mg/5ml
S, T	172	cloxacillin + ampicillin	powder for suspension	250mg/5ml

S2: Category wise List of essential medicines with color-coding and result summary

**WATCH GROUP ANTI- BIOTICS**				
S, T	173	ceftriaxone	powder for injection	250mg
S, T	174	ceftriaxone	powder for injection	500mg
S, T	175	ceftriaxone	powder for injection	1g
S, T	176	cefixime	capsules	400mg
S, T	177	cefixime	suspension	100mg/5ml
S, T	178	cefixime	suspension	200mg/5ml
S, T	179	piperacillin + tazobactum	powder for injection	2g + 250mg
S, T	180	piperacillin + tazobactum	powder for injection	4g + 500mg
S, T	181	ceftazidime	powder for injection	250mg
S, T	182	ceftazidime	powder for injection	500mg
S, T	183	ceftazidime	powder for injection	1g
T	184	meropenum	injection	500mg
**6.2.2 OTHER ANTI-BACTERIALS**				
T	185	chloramphenicol	capsules	250mg
T	186	chloramphenicol**	oral liquid	150mg/5ml *125mg/5ml
T	187	chloramphenicol	powder for injection	1g
T	188	amikacin (sulphate)	injection	100mg
T	189	amikacin (sulphate)	injection	250mg
S, T	190	clindamycin	capsules	150mg
S, T	191	clindamycin	capsules	300mg
S, T	192	clindamycin	injection	150mg/ml
S, T	193	clindamycin	oral liquid	75mg/5ml
P, S, T	194	doxycycline	oral liquid	25mg/5ml
P, S, T	195	doxycycline	oral liquid	50mg/5ml
P, S, T	196	doxycycline	solid oral dosage form	50mg
P, S, T	197	doxycycline	solid oral dosage form	100mg
S, T	198	ciprofloxacin	oral liquid	250mg/5ml
S, T	199	ciprofloxacin	solution for I/V infusion	2mg/ml
S, T	200	ciprofloxacin	tablet (coated)	250mg
S, T	201	ciprofloxacin	tablet (coated)	500mg
S, T	202	moxifloxacin	tablet	200mg
S, T	203	moxifloxacin	tablet	400mg
P, S, T	204	azithromycin	capsules	250mg
P, S, T	205	azithromycin	capsules	500mg

P, S, T	206	azithromycin	oral liquid	200mg/5ml
S, T	207	clarithromycin	oral liquid	125mg/5ml
S, T	208	clarithromycin	solid oral dosage form	500mg
P, S, T	209	doxycycline	oral liquid	25mg/5ml
P, S, T	210	doxycycline	oral liquid	50mg/5ml
P, S, T	211	doxycycline	solid oral dosage form	50mg
P, S, T	212	doxycycline	solid oral dosage form	100mg
P, S, T	213	gentamicin	injection	10mg/ml in 2ml vial
P, S, T	214	gentamicin	injection	40mg/ml in 2ml vial
P, S, T	215	metronidazole	injection	500mg in 100ml vial
P, S, T	216	metronidazole	oral liquid	200mg/5ml
P, S, T	217	metronidazole	tablet	400mg
P, S, T	218	nitrofurantoin	oral liquid	25mg/5ml
P, S, T	219	nitrofurantoin	tablet	100mg
P, S, T	220	sulfamethoxazole + trimethoprim	injection	80mg + 16mg/ml in 5ml ampoule
P, S, T	221	sulfamethoxazole + trimethoprim	injection	80mg + 16mg/ml in 10ml ampoule
P, S, T	222	sulfamethoxazole + trimethoprim	oral liquid	200mg + 40mg/5ml
P, S, T	223	sulfamethoxazole + trimethoprim	tablet	100mg + 20mg
P, S, T	224	sulfamethoxazole + trimethoprim	tablet	400mg + 80mg
P, S, T	225	sulfamethoxazole + trimethoprim	tablet	800mg + 160mg
T	226	spectinomycin	powder for injection	2g in vial
T	227	vancomycin	powder for injection	250mg in vial
T	228	vancomycin	powder for injection	500mg in vial
T	229	aztreonam	injection	500mg in vial
T	230	aztreonam	injection	1g in vial
**6.2.3 ANTI-TUBERCULOSIS MEDICINES**				
NTP GUIDELINES	231	ethambutol	oral liquid	25mg/ml
NTP GUIDELINES	232	ethambutol	tablet	100mg -400mg
NTP GUIDELINES	233	ethambutol + isoniazid	tab	400mg + 150mg

S2: Category wise List of essential medicines with color-coding and result summary

NTP GUIDELINES	234	ethambutol + isoniazid + pyrazinamide + rifampicin	tablet	275mg + 75mg + 400mg + 150mg
NTP GUIDELINES	235	ethambutol + isoniazid + rifampicin	tablet	275mg + 75mg + 150mg
NTP GUIDELINES	236	isoniazid	oral liquid	50mg/5ml
NTP GUIDELINES	237	isoniazid	tablet	100mg to 300mg
NTP GUIDELINES	238	isoniazid	tablet (scored)	50mg
NTP GUIDELINES	239	isoniazid + pyrazinamide + rifampicin	tablet	75mg + 400mg + 150mg
NTP GUIDELINES	240	isoniazid + pyrazinamide + rifampicin	tablet	150mg + 500mg + 150mg
NTP GUIDELINES	241	isoniazid + rifampicin	tablet	75mg + 150mg *100mg + 150mg
NTP GUIDELINES	242	isoniazid + rifampicin	tablet	150mg + 300mg
NTP GUIDELINES	243	isoniazid + rifampicin	tablet	60mg + 60mg (for intermittent use three times a week)
NTP GUIDELINES	244	isoniazid + rifampicin	tablet	150mg + 150mg (for intermittent use three times a week)
NTP GUIDELINES	245	pyrazinamide	oral liquid	30mg/ml * 50mg/ml
NTP GUIDELINES	246	pyrazinamide	tablet	400mg * 500mg
NTP GUIDELINES	247	pyrazinamide	tablet (dispersible)	150mg
NTP GUIDELINES	248	pyrazinamide	tablet (scored)	150mg
NTP GUIDELINES	249	rifabutin	capsules	150mg
NTP GUIDELINES	250	rifampicin	oral liquid	20mg/ml
NTP GUIDELINES	251	rifampicin	solid oral dosage form	150mg
NTP GUIDELINES	252	rifampicin	solid oral dosage form	300mg
NTP GUIDELINES	253	rifapentine	tablet	150mg
NTP GUIDELINES	254	streptomycin	powder for injection	1g
S, T	255	amikacin	powder for injection	100mg
S, T	256	amikacin	powder for injection	500mg
S, T	257	amikacin	powder for injection	1g

NTP GUIDELINES	258	bedaquiline	tablet	100mg
NTP GUIDELINES	259	capreomycin	powder for injection	1g in vial
NTP GUIDELINES	260	cycloserine	solid oral dosage form	250mg
NTP GUIDELINES	261	delamanid	tablet	50mg
NTP GUIDELINES	262	ethionamide	tablet	125mg
NTP GUIDELINES	263	ethionamide	tablet	250mg
NTP GUIDELINES	264	kanamycin	powder for injection	1g
S,T	265	levofloxacin	tablet	250mg
S,T	266	levofloxacin	tablet	500mg
S,T	267	levofloxacin	tablet	750mg
NTP GUIDELINES	268	linezolid	injection for I/V administration	2mg/ml in 300ml bag
NTP GUIDELINES	269	linezolid	powder for oral liquid	100mg/5ml
NTP GUIDELINES	270	linezolid	tablet	400mg
NTP GUIDELINES	271	linezolid	tablet	600mg
NTP GUIDELINES	272	p-aminosalicylic acid	granules	4gm in sachet
NTP GUIDELINES	273	p-aminosalicylic acid	tablet	500mg
NTP GUIDELINES	274	clofazimine	capsules	50mg
NTP GUIDELINES	275	clofazimine	capsules	100mg
**6.3 ANTI-FUNGAL MEDICINES**				
S, T	276	amphoteracin B	powder for injection	50mg in vial
P, S, T	277	clotrimazole	vaginal cream	1% (mg)
P, S, T	278	clotrimazole	vaginal cream	10% (mg)
P, S, T	279	clotrimazole	vaginal tablet	100mg
P, S, T	280	clotrimazole	vaginal tablet	500mg
P, S, T	281	fluconazole	capsules	50mg
P, S, T	282	fluconazole	capsules	150mg
P, S, T	283	fluconazole	capsules	200mg
S, T	284	fluconazole	injection	2mg/ml
P, S, T	285	fluconazole	oral liquid	50mg/5ml
P, S, T	286	flucytosine	capsules	250mg
P, S, T	287	flucytosine	infusion	2.5g in 250ml
P, S, T	288	griseofulvin	oral liquid	125mg/5ml
P, S, T	289	griseofulvin	solid oral dosage form	125mg

P, S, T	290	griseofulvin	solid oral dosage form	250mg
P, S, T	291	nystatin	lozenge	100 000 IU
P, S, T	292	nystatin	oral liquid	50mg/5ml
P, S, T	293	nystatin	oral liquid	100, 000 IU/ml
P, S, T	294	nystatin	pessary	100,000 IU
P, S, T	295	nystatin	tablet	100, 000 IU
P, S, T	296	nystatin	tablet	500, 000 IU
S, T	297	itraconazole	capsules	100mg
S, T	298	itraconazole	oral liquid	10mg/ml
**6.4 ANTI-VIRAL MEDICINES**				
**6.4.1 ANTI-HERPES MEDICINES**				
P, S, T	299	aciclovir	oral liquid	200mg/5ml
P, S, T	300	aciclovir	powder for injection	250mg
P, S, T	301	aciclovir	tablet	200mg
**6.4.2.1 NUCLEOSIDE/NUCLEOTIDE REVERSE TRANSCRIPTASE INHIBITORS**				
S, T	302	abacavir	oral liquid	100mg/5ml
S, T	303	lamivudine	oral liquid	50mg/5ml
S, T	304	lamivudine	tablet	150mg
S, T	305	entecavir	tablet	0.5mg
S, T	306	entecavir	tablet	1mg
S, T	307	zidovudine (ZDV or AZT)	capsules	100mg
S, T	308	zidovudine (ZDV or AZT)	oral liquid	50mg/5ml
S, T	309	zidovudine (ZDV or AZT)	solution for I/V infusion	10mg/ml in 20ml vial
S, T	310	zidovudine (ZDV or AZT)	tablet	300mg
**6.4.2.2 NON-NUCLEOSIDE REVERSE TRANSCRIPTASE INHIBITORS**				
S, T	311	efavirenz	tablet	200mg
S, T	312	efavirenz	tablet	600mg
S, T	313	nevirapine	tablet	50mg
S, T	314	nevirapine	tablet	200mg
**6.4.2.3 PROTEASE INHIBITORS**				
S, T	315	atazanavir	solid oral dosage form	100mg
S, T	316	atazanavir	solid oral dosage form	150mg
S, T	317	atazanavir	solid oral dosage form	300mg
S, T	318	atazanavir + ritonavir	tablet	300mg + 100mg
S, T	319	darunavir	tablet	75mg
S, T	320	darunavir	tablet	400mg

S, T	321	darunavir	tablet	600mg
S, T	322	darunavir	tablet	800mg
S, T	323	ritonavir	oral liquid	400mg/5ml
S, T	324	ritonavir	tablet (heat stable)	25mg
S, T	325	ritonavir	tablet (heat stable)	100mg
**6.4.3 OTHER ANTI-VIRALS**				
T	326	dolutegravir	tablet (Heat stable)	50mg
T	327	raltegravir	tablet (heat stable)	400mg
T	328	isoniazid + pyridoxine + sulfamethoxazole + trimethoprim	tablet	300mg + 25mg + 800mg + 160mg
S, T	329	ribavirin	injection for I/V administration	800mg in 10ml phosphate buffer solution
S, T	330	ribavirin	injection for I/V administration	1g in 10ml phosphate buffer solution
T	331	ribavirin	solid oral dosage form	200mg
T	332	ribavirin	solid oral dosage form	400mg
T	333	ribavirin	solid oral dosage form	600mg
**6.4.4 ABNTI-HEPATITIS MEDICINES**				
**6.4.4.1 MEDICINES FOR HEPATITIS B**				
S, T	334	entecavir	oral liquid	0.05mg/ml
S, T	335	entecavir	tablet	0.5mg
S, T	336	entecavir	tablet	1mg
**6.4.4.1.1 NUCLEOSIDE/NUCLEOTIDE REVERSE TRANSCRIPTASE INHIBITORS**				
S, T	337	tenofovir disoproxil fumarate	tablet	300mg (TDF equivalent to 245mg tenofovir disoproxil)
**6.4.4.2 MEDICINES FOR HEPATITIS C**				
**6.4.4.2.1 NUCLEOTIDE POLYMERASE INHIBITORS**				
S, T	338	sofosbuvir	tablet	400mg
**6.4.4.2.2 PROTEASE INHIBITOTRS**				
S, T	339	simeprevir	capsule	150mg
**6.4.4.2.3 NS5A INHIBITORS**				
S, T	340	daclatasvir	tablet	30mg
S, T	341	daclatasvir	tablet	60mg
**6.4.4.2.4 NON-NUCLEOSIDE POLYMERASE INHIBITORS**				

S, T	342	Dasabuvir	tablet	250mg
**6.4.4.2.5 OTHER ANTI-VIRALS**				
S, T	343	ribavirin	injection for I/V administration	800mg in 10ml phosphate buffer solution
S, T	344	ribavirin	injection for I/V administration	1g in 10ml phosphate buffer solution
S, T	345	ribavirin	solid oral dosage form	200mg
S, T	346	ribavirin	solid oral dosage form	400mg
S, T	347	ribavirin	solid oral dosage form	600mg
T	348	pegylated interferon alfa (2a)	vial or pre-filled syringe	180 micrograms
T	349	pegylated interferon alfa (2b)	vial or pre-filled syringe	80 micrograms
T	350	pegylated interferon alfa (2b)	vial or pre-filled syringe	100 micrograms
T	351	ledipasvir + sofosbuvir	tablet	90mg + 400mg
T	352	ombitasvir + paritapvir + ritonavir	tablet	12.5mg + 75mg + 50mg
**6.5 ANTI-PARASITICS**				
**6.5.1 ANTI-AMOEBIC AND ANTI-GIARDIASIS MEDICINES**				
P, S, T	353	diloxanide	tablet	500mg
P, S, T	354	metronidazole	injection	50mg in 100ml vial
P, S, T	355	metronidazole	oral liquid	200mg/5ml
P, S, T	356	metronidazole	tablet	400mg
**6.5.2 ANTI-LEISHMINIASIS MEDICINES**				
S, T	357	amphoteracin B	Powder for injection	50mg in vial
P, S, T	358	miltefosine	solid oral dosage form	10mg
P, S, T	359	miltefosine	solid oral dosage form	50mg
P, S, T	360	paromomycin	solution for I/M injection	750mg of parmomycin
S, T	361	meglumine anti-moniate	injection	30 %, equivalent to approx. 8.1 % antimony in 5ml ampoule
S, T	362	sodium stibogluconate	injection	100mg/ml, 1 vial = 30ml
**6.5.3 ANTI-MALARIAL MEDICINES**				
MALARIA CONTROL PROGRAMME	363	artemether + lumefantrine	tablet	20mg + 120mg

MALARIA CONTROL PROGRAMME	364	artemether + lumefantrine	tablet (dispersible)	20mg + 120mg
MALARIA CONTROL PROGRAMME	365	artesunate	injection	ampoules containing 60mg anhydrous artesunic acid with separate ampoule of 5% sodium bicarbonate
MALARIA CONTROL PROGRAMME	366	artesunate	rectal dosage form	50mg
MALARIA CONTROL PROGRAMME	367	artesunate	rectal dosage form	200mg
MALARIA CONTROL PROGRAMME	368	artesunate	tablet	50mg
MALARIA CONTROL PROGRAMME	369	primaquine	tablet	7.5mg
MALARIA CONTROL PROGRAMME	370	primaquine	tablet	15mg
MALARIA CONTROL PROGRAMME	371	artesunate + sulphadoxine and pyrimethamine	co-blister of 6+2 tablets	sulfadoxine 500mg + pyrimethamine 25mg and artesunate 50mg
MALARIA CONTROL PROGRAMME	372	artesunate + sulphadoxine and pyrimethamine	co-blister of 6+3 tablets	sulfadoxine 500mg + pyrimethamine 25mg and artesunate 100mg
MALARIA CONTROL PROGRAMME	373	sulphadoxine + pyrimethamine (co-blister / combined therapy)	tablets	500mg + 25mg
**6.5.3.2 FOR PROPHYLAXIS**				
P, S, T	374	chloroquine	oral liquid	50mg/5ml
P, S, T	375	chloroquine	tablet	150mg
P, S, T	376	doxycycline	solid oral dosage form	100mg
MALARIA CONTROL PROGRAMME	377	mefloquine	tablet	250mg
MALARIA CONTROL PROGRAMME	378	proguanil	tablet	100mg

MALARIA CONTROL PROGRAMME	379	dihydroartimisinin + piperquine	tablet	320 mg + 40mg
MALARIA CONTROL PROGRAMME	380	dihydroartimisinin + piperquine	tablet	160mg + 20mg
**Generics**	**Expanded medicinal products**	**Unregistered medicinal products (Yellow)**	**Medicinal products with slightly different specifications (Orange)**	**Unregistered medicines can be available through compounding**
**111**	**254**	**50**	**6**	**13**

**NEML-2018**				
**7. ANTI-MIGRAINE MEDICINES**				
**SECTOR**	**UID**	**MEDICINE**	**DOSAGE FORM**	**STRENGTH**
**FOR TREATMENT OF ACCUTE ATTACK**				
**P, S, T**	381	acetylsalicylic acid	tablet	300mg to 500mg
**P, S, T**	382	ibuprofen	tablet	200mg
**P, S, T**	383	ibuprofen	tablet	400mg
**P, S, T**	384	paracetamol	oral liquid	125mg/5ml
**P, S, T**	385	paracetamol	tablet	300mg to 500mg
**S, T**	386	cinnarizine	tablet	25mg
**S, T**	387	sumatriptan	injection	6mg
**S, T**	388	sumatriptan	tablets	50mg
**FOR PROPHYLAXIS**				
**P, S, T**	389	Propranolol	tablet	20mg
**P, S, T**	390	Propranolol	tablet	40mg
**Generics**	**Expanded medicinal products**	**Unregistered medicinal products (Yellow)**	**Medicinal products with slightly different specifications (Orange)**	**Unregistered medicines can be available through compounding**
**6**	**10**	**1**	**0**	**0**

NEML-2018				
**9. ANTI-PARKINSONISM MEDICINES**				
**SECTOR**	**UID**	**MEDICINE**	**DOSAGE FORM**	**STRENGTH**
**S, T**	391	Biperiden	injection	5mg in 1ml ampoule
**S, T**	392	Biperiden	tablet	2mg
**Generics**	**Expanded medicinal products**	**Unregistered medicinal products (Yellow)**	**Medicinal products with slightly different specifications (Orange)**	**Unregistered medicines can be available through compounding**
**1**	**2**	**2**	**0**	**0**

NEML-2018				
**10. MEDICINES AFFECTING THE BLOOD**				
**SECTOR**	**UID**	**MEDICINE**	**DOSAGE FORM**	**STRENGTH**
**10.1 ANTI-ANAEMIA MEDICINES**				
P, S, T	393	ferrous salt**	oral liquid	equivalent to 25mg iron *50mg iron
P, S, T	394	ferrous salt	tablet	equivalent to 60mg iron *65mg
P, S, T	395	ferrous salt + folic acid	tablet	equivalent to 60mg iron + 400 mcg folic acid *100mg + 500mcg
P, S, T	396	folic acid	tablet	400mcg
P, S, T	397	folic acid	tablet	1mg
P, S, T	398	folic acid	tablet	5mg
P, S, T	399	hydroxocobalamin	injection	1mg in 1ml ampule
T	400	tirofiban	injection	0.25mg/ml
S, T	401	iron sucrose	injection	20mg/ml
**10.2 MEDICINES AFFECTING COAGULATION**				
T	402	enoxaparin	injection	20mg/0.2ml
T	403	enoxaparin	injection	40mg/0.4ml
T	404	enoxaparin	injection	60mg/0.6ml
T	405	enoxaparin	injection	80mg/0.8ml
T	406	enoxaparin	injection	100mg/1ml
T	407	enoxaparin	injection	120mg/0.8ml
T	408	enoxaparin	injection	150mg/1ml
S, T	409	clopidogrel	tablet	75mg
S, T	410	heparin sodium	injection	1000IU/ml

S, T	411	heparin sodium	injection	5000IU/ml
S, T	412	heparin sodium	injection	20,000IU/ml in 1ml ampule *25,000IU/ml
P, S, T	413	phytomenadione	injection	1mg/ml ampoule *2mg/ml
P, S, T	414	phytomenadione	injection	10mg/ml in 5ml
P, S, T	415	phytomenadione	tablet	10mg
S, T	416	protamine sulfate	injection	10mg/ml in 5ml ampoule
P, S., T	417	tranexamic acid	injection	100mg/ml in 10ml ampoule
P, S., T	418	tranexamic acid	capsules	250mg
P, S., T	419	tranexamic acid	capsules	500mg
S, T	420	warfarin	tablet	1mg
S, T	421	warfarin	tablet	2mg
S, T	422	warfarin	tablet	5mg
T	423	desmopressin	injection	4mcg/ml in 1ml ampoule
T	424	desmopressin	nasal spray	10mcg/dose
**10.3 OTHER MEDICINES FOR HEAMOGLOBINOPATHIES**				
S, T	425	deferoxamine	powder for injection	500mg in vial
S, T	426	hydroxycarbamide	solid oral dosage form	200mg
S, T	427	hydroxycarbamide	solid oral dosage form	500mg
S, T	428	hydroxycarbamide	solid oral dosage form	1gm
**Generics**	**Expanded medicinal products**	**Unregistered medicinal products (Yellow)**	**Medicinal products with slightly different specifications (Orange)**	**Unregistered medicines can be available through compounding**
**16**	**36**	**4**	**6**	**0**

S2: Category wise List of essential medicines with color-coding and result summary

NEML-2018				
12+16 CARDIOVASCULAR MEDICINES+DIURETICS				
SECTOR	UID	MEDICINE	DOSAGE FORM	STRENGTH
12.1 ANTI-ANGINAL MEDICINES				
P, S, T	429	atenolol	tablet	50mg
P, S, T	430	atenolol	tablet	100mg
P, S, T	431	glyceryl trinitrate	tablet (sub-lingual)	500mcg
P, S, T	432	isosorbide dinitrate	tablet (sub-lingual)	5mg
S	433	losartan potassium	tablet	50mg
S	434	propranolol	tablets	10mg
S	435	propranolol	tablets	40mg
S	436	propranolol	tablets	80mg
S, T	437	verapamil	tablet	40mg
S, T	438	verapamil	tablet	80mg
12.2 ANTIARRHYTHMIC MEDICINES				
P, S, T	439	bisoprolol	tablet	1.25mg
P, S, T	440	bisoprolol	tablet	5mg
S, T	441	digoxin	injection	250 mcg/ml in 2-ml ampule
S, T	442	digoxin	oral liquid	50mcg/ml
S, T	443	digoxin	tablet	62.5 mcg
S, T	444	digoxin	tablet	250mcg
P, S, T	445	epinephrine(adrenaline)	injection	100mcg/ml in 10ml ampule
P, S, T	446	lidocaine	injection	20mg/ml in 5ml ampule
S, T	447	verapamil	injection	2.5mg/ml in 2ml ampule
S, T	448	verapamil	tablet	40mg
S, T	449	verapamil	tablet	80mg
S, T	450	amiodarone	injection	50mg/ml in 3ml ampule
S, T	451	amiodarone	tablet	100mg
S, T	452	amiodarone	tablet	200mg
S, T	453	amiodarone	tablet	400mg
S, T	454	adenosine	injection	3mg/ml
P, S, T	455	labetalol	injection	5mg/ml
12.3 ANTIHYPERTENSIVE MEDICINES				
P, S, T	456	amlodipine	tablet	5mg
P, S, T	457	bisoprolol	tablet	1.25mg
P, S, T	458	bisoprolol	tablet	5mg

P, S, T	459	propranolol	tablet	10mg
P, S, T	460	propranolol	tablet	40mg
P, S, T	461	propanalol	tablet	80mg
S, T	462	hydralazine	injection	20mg
P, S, T	463	enalapril	tablet	2.5mg
P, S, T	464	enalapril	tablet	5mg
S, T	465	hydralazine	powder for injection	20mg
S, T	466	hydralazine	tablet	25mg
S, T	467	hydralazine	tablet	50mg
S, T	468	hydrochlorothiazide	oral liquid	50mg/5ml
P, S, T	469	hydrochlorothiazide	solid oral dosage form	12.5mg
P, S, T	470	hydrochlorothiazide	solid oral dosage form	25mg
P, S, T	471	hydrochlorothiazide	injection	20mg
P, S, T	472	methyldopa	tablet	250mg
P, S, T	473	methyldopa	injection	250mg
T	474	sodium nitroprusside	powder for infusion	50mg in ampule
T	475	isoprenaline	injection	1mg/ml
**12.4 MEDICINES USED IN HEART FAILURE**				
P, S, T	476	bisoprolol	tablet	1.25mg
P, S, T	477	bisoprolol	tablet	5mg
S, T	478	digoxin	injection	250mcg/ml in 2ml ampule
S, T	479	digoxin	oral liquid	50mcg/ml
S, T	480	digoxin	tablet	62.5mcg
S, T	481	digoxin	tablet	250mcg
P, S, T	482	enalapril	tablet	2.5mg
P, S, T	483	enalapril	tablet	5mg
S, T	484	enalapril	injection	10mg/ml
P, S, T	485	frusemide	oral liquid	20mg/5ml or 4mg/ml
P, S, T	486	furosemide	tablet	40mg
P, S, T	487	hydrochlorothiazide	oral liquid	50mg/5ml
P, S, T	488	hydrochlorothiazide	solid oral dosage form	25mg
P, S, T	489	spironolactone	tablet	25mg
S, T	490	dopamine	injection	40mg/ml in 5ml vial
S, T	491	dobutamine	injection	250mg
S, T	492	ephedrine	injection	30mg/ml
**12.5 ANTITHROMBOTIC MEDICINES**				
**12.5.1 ANTI-PLATELET MEDICINES**				
P, S, T	493	acetylsalicylic acid	tablet	100mg

T	494	clopidogeral	tablet	75mg
T	495	clopidogeral	tablet	300mg
**12.5.2 THROMBOTIC MEDICINES**				
S, T	496	streptokinase	powder for injection	1.5 million IU in vial
**12.6 LIPID-LOWERING AGENTS**				
T	497	simvastatin	tablet	5mg
T	498	simvastatin	tablet	10mg
T	499	simvastatin	tablet	20mg
T	500	simvastatin	tablet	40mg
S, T	501	atorvastatin	tablet	10mg
S, T	502	atorvastatin	tablet	20mg
S, T	503	atorvastatin	tablet	40mg
S, T	504	risovastatin	tablet	10mg
S, T	505	risovastatin	tablet	20mg
S, T	506	risovastatin	tablet	40mg
**DIURETICS**				
S, T	507	amiloride	tablet	5mg
P, S, T	508	furosemide	injection	10mg/ml in 2ml ampule
P, S, T	509	furosemide	oral liquid	20mg/5ml
P, S, T	510	furosemide	tablet	10mg
P, S, T	511	furosemide	tablet	20mg
P, S, T	512	furosemide	tablet	40mg
P, S, T	513	hydrochlorothiazide	solid oral dosage form	25mg
P, S, T	514	hydrochlorothiazide	injection	20mg
P, S, T	515	mannitol	injectable solution	10% solution
P, S, T	516	mannitol	injectable solution	20% solution
P, S, T	517	spironolactone	oral liquid	5mg/5ml
P, S, T	518	spironolactone	oral liquid	10mg/5ml
P, S, T	519	spironolactone	oral liquid	25mg/5ml
P, S, T	520	spironolactone	tablet	25mg
T	521	sodium polystyrene sulfonate	powder	
**Generics**	**Expanded medicinal products**	**Unregistered medicinal products (Yellow)**	**Medicinal products with slightly different specifications (Orange)**	**Unregistered medicines can be available through compounding**
**39**	**78**	**9**	**0**	**3**
**6**	**15**	**2**	**0**	**5**

NEML-2018				
**17. Gastrointestinal Medicines**				
**SECTOR**	**UID**	**MEDICINE**	**DOSAGE FORM**	**STRENGTH**
**T**	522	pancreatic enzymes	age appropriate formulation	
**17.1 ANTI-ULCER MEDICINES**				
**P, S, T**	523	omeprazole	powder for injection	40mg in vial
**P, S, T**	524	omeprazole	powder for oral liquid	20mg
**P, S, T**	525	omeprazole	powder for oral liquid	40mg
**P, S, T**	526	omeprazole	solid oral dosage form	10mg
**P, S, T**	527	omeprazole	solid oral dosage form	20mg
**P, S, T**	528	omeprazole	solid oral dosage form	40mg
**P, S, T**	529	ranitidine	injection	25mg/ml in 2ml ampoule
**P, S, T**	530	ranitidine	oral liquid	75mg/5ml
**P, S, T**	531	ranitidine	tablet	150mg
**17.2 ANTI-EMETIC MEDICINES**				
**P, S, T**	532	dexamethasone	injection	4mg/ml in 1ml ampoule
**P, S, T**	533	dexamethasone	oral liquid	0.5mg/5ml
**P, S, T**	534	dexamethasone	oral liquid	2mg/5ml
**P, S, T**	535	dexamethasone	solid oral dosage form	0.5mg
**P, S, T**	536	dexamethasone	solid oral dosage form	0.75mg
**P, S, T**	537	dexamethasone	solid oral dosage form	1.5mg
**P, S, T**	538	dexamethasone	solid oral dosage form	4mg
**P, S, T**	539	metoclopramide	injection	5mg /ml in 2ml ampoule
**P, S, T**	540	metoclopramide	oral liquid	5mg/5ml
**P, S, T**	541	metoclopramide	tablet	10mg
**S, T**	542	ondansteron	injection	2mg base/ml in 2ml ampoule
**S, T**	543	ondansteron	oral liquid	4mg base/5ml
**S, T**	544	ondansteron	solid oral dosage form	equivalent to 4 mg base
**S, T**	545	ondansteron	solid oral dosage form	equivalent to 8 mg base

**S, T**	546	ondansteron	solid oral dosage form	equivalent to 24 mg base
**17.3 ANTI-INFLAMMATORY MEDICINES**				
**S, T**	547	sulfasalazine	retention enema	
**P, S, T**	548	sulfasalazine	suppository	500mg
**P, S, T**	549	sulfasalazine	tablet	500mg
**S, T**	550	hydrocortisone	retention enema	
**S, T**	551	hydrocortisone	suppository	25mg
**17.4 LAXATIVES**				
**P, S, T**	552	senna	tablet	7.5mg
**17.5 MEDICINES USED IN DIARRHEA**				
**P, S, T**	553	ORS	dry mixture in sachet	For 1 liter solution
**P, S, T**	554	zinc sulfate	solid oral dosage form	20mg
**17.6 OTHER MEDICINES**				
**P, S, T**	555	aluminium hydroxide + magnesium trisilicate	suspension	215mg + 80mg/5ml
**P, S, T**	556	bisacodyl	tablet	5mg
**P, S, T**	557	dimenhydrinate	injection	40mg in vial *50mg
**P, S, T**	558	dimenhydrinate	tablet	50mg
**P, S, T**	559	glycerin	suppository	
**P, S, T**	560	magnesium oxides and hydroxides	suspension	7.9% w/v
**P, S, T**	561	phloroglucinol	injection	10mg/ml
**P, S, T**	562	sodium biphosphate	enema	7.2g/120ml
**Generics**	**Expanded medicinal products**	**Unregistered medicinal products (Yellow)**	**Medicinal products with slightly different specifications (Orange)**	**Unregistered medicines can be available through compounding**
18	41	3	1	6

S2: Category wise List of essential medicines with color-coding and result summary

NEML-2018				
**18. HORMONES, OTHER ENDOCRINE MEDICINES AND CONTRACEPTIVES**				
**SECTOR**	**UID**	**MEDICINE**	**DOSAGE FORM**	**STRENGTH**
**18.1 ADRENAL HORMONES AND SYNTHETIC SUBSTITUTE**				
T	563	fludrocortisone	tablet	100mcg
S, T	564	hydrocortisone	tablet	5mg
S, T	565	hydrocortisone	tablet	10mg
S, T	566	hydrocortisone	tablet	20mg
**18.2 ANDROGENS**				
S, T	567	testosteron	injection	200mg in 1ml ampule *250mg/ml
**CONTRACEPTIVES**				
**18.3.1 ORAL HORMONAL CONTRACEPTIVES**				
P, S, T	568	ethinylestradiol + levonorgestrel	tablet	30mcg + 150mcg
P, S, T	569	ethinylestradiol + norethisterone	tablet	35mcg + 1mg *20mcg + 10mg
P, S, T	570	levonorgestrel	tablet	30mcg
P, S, T	571	levonorgestrel	tablet	750mcg
P, S, T	572	levonorgestrel	tablet	1.5mg
**18.3.2 INJECTABLE HORMONAL CONTRACEPTIVES**				
S, T	573	estradiol cypionate + medroxyprogesteron acetate	injection	5mg + 25mg
S, T	574	medroxyprogesteron acetate	depot injection	150mg/ml in 1 ml vial
S, T	575	norethisterone enantate	oily solution	200mg/ml in 1ml ampoule
**18.3.3 INTRAUTERINE DEVICES**				
P, S, T	576	copper containing devices		
T	577	levonorgestrel-releasing intrauterine system	intrauterine reservoir	52mg of levonorgestrel
**18.3.4 BARRIER METHODS**				
P, S, T	578	condoms		
P, S, T	579	diaphragm		
S, T	580	etonogestrel-releasing implant	single rod etonogestrel-releasing implant	68 mg of etonogestrel
**18.3.5 IMPLANTABLE CONTRACEPTIVES**				
S, T	581	levonorgestrel-releasing plant	two rod levonorgestrel-releasing implant	each contains 75mg total 150mg *52mg
**18.3.6 INTRAVAGINAL CONTRACEPTIVES**				

S, T	582	progesterone vaginal ring	progesterone-releasing vaginal ring	2.074 grams of micronized progesterone
**18.4 ESTROGEN**				
S, T	583	estrogen	estrogen	
**18.5 INSULIN AND OTHER MEDICINES USED FOR DIABETES**				
S, T	584	gliclazide	solid oral dosage form (controlled release tablets)	30mg
S, T	585	gliclazide	solid oral dosage form (controlled release tablets)	60mg
S, T	586	gliclazide	solid oral dosage form (controlled release tablets)	80mg
T	587	glucagon	injection	1mg/1ml
P, S, T	588	insulin injection (soluble)	injection	40 IU/ml in 10ml vial
P, S, T	589	insulin injection (soluble)	injection	100IU /ml in 10ml vial
P, S, T	590	intermediate acting insulin	injection	40IU/ml in 10ml vial
P, S, T	591	intermediate acting insulin	injection	100IU /ml in 10ml vial
P, S, T	592	metformin	tablet	500mg
P, S, T	593	glibenclamide	tablet	5mg
**18.6 OVULATION INDUCER**				
S, T	594	clomifene	tablet	50mg
**18.7 PROGESTOGEN**				
P, S, T	595	medroxyprogesteron	tablet	5mg acetate *500mg
**18.8 THYROID HORMONE AND ANTI-THYROID MEDICINES**				
P, S, T	596	levothyroxine	tablet	25mcg
P, S, T	597	levothyroxine	tablet	50mcg
P, S, T	598	levothyroxine	tablet	100mcg
P, S, T	599	potassium iodide	tablet	60mg *130mg
S, T	600	propylthiouracil	tablet	50mg
P, S, T	601	lugol's solution	oral liquid	about 130mg total iodine/ml
P, S, T	602	propylthiouracil	tablet	50mg
**Generics**	**Expanded medicinal products**	**Unregistered medicinal products (Yellow)**	**Medicinal products with slightly different specifications (Orange)**	**Unregistered medicines can be available through compounding**
30	40	9	5	1

S2: Category wise List of essential medicines with color-coding and result summary

NEML-2018				
20. MUSCLES RELAXANTS				
Sector	UID	MEDICINE	DOSAGE FORM	STRENGTH
S, T	603	atracurium	injection	10mg/ml
S, T	604	neostigmine	injection	500mcg in 1ml ampoule
S, T	605	neostigmine	injection	2.5mg in 1ml ampoule
S, T	606	suxamethonium	injection	50mg /ml in 2ml ampoule
S, T	607	suxamethonium	powder for injection	
P, S, T	608	vecuronium	powder for injection	10mg in vial
S, T	609	pyridostigmine	injection	1mg in 1ml ampoule *0.5mg/ml
S, T	610	pyridostigmine	tablet	60mg
S, T	611	pancuronium	injection	2mg/ml, 2ml
**Generics**	**Expanded medicinal products**	**Unregistered medicinal products (Yellow)**	**Medicinal products with slightly different specifications (Orange)**	**Unregistered medicines can be available through compounding**
**6**	**9**	**0**	**1**	**0**

NEML-2018				
22. OXYTOCICS AND ANTI-OXYTOCICS				
SECTOR	UID	MEDICINE	DOSAGE FORM	STRENGTH
22.1 OXYTOCICS				
P,S,T	612	ergometrine	injection	200mcg in 1ml ampoule *500mcg/ml
ST	613	mesoprostol	tablet	200mcg
ST	614	mesoprostol	vaginal tablet	25mcg
ST	615	oxytocin	injection	10 IU in 1ml
22.2 ANTI-OXYTOCICS				
S,T	616	nifedipine	capsules (immediate release)	10mg
T	617	nifedipine	tablet (slow release)	20mg
**Generics**	**Expanded medicinal products**	**Unregistered medicinal products (Yellow)**	**Medicinal products with slightly different specifications (Orange)**	**Unregistered medicines can be available through compounding**
**4**	**6**	**0**	**1**	**0**

NEML-2018				
**24. MEDICINES FOR MENTAL AND BEHAVIORAL DISORDERS**				
**SECTOR**	**UID**	**MEDICINE**	**DOSAGE FORM**	**STRENGTH**
**24.1 MEDICINES USED IN PSYCHOTIC DISORDERS**				
P, S, T	618	chlorpromazine	injection	25mg/ml in 2ml ampoule
P, S, T	619	chlorpromazine	oral liquid	25mg/5ml
P, S, T	620	chlorpromazine	tablet	10mg
P, S, T	621	chlorpromazine	tablet	25mg
P, S, T	622	chlorpromazine	tablet	50mg
P, S, T	623	chlorpromazine	tablet	100mg
S, T	624	fluphenazine	injection	25mg in 1ml ampoule
S, T	625	haloperidol	injection	5mg in 1ml ampoule
S, T	626	haloperidol	oral liquid	2mg/ml
S, T	627	haloperidol	tablet	0.5mg
S, T	628	haloperidol	tablet	2mg
S, T	629	haloperidol	tablet	5mg
S, T	630	risperidone	solid oral dosage form	0.25mg to 6.0mg
S, T	631	clozapine	solid oral dosage form	25 to 200mg
**24.2 MEDICINES USED IN MOOD DISORDERS**				
**24.2.1. MEDICINES USED IN DEPRESSIVE DISORDERS**				
P, S, T	632	amitriptyline	tablet	10mg
P, S, T	633	amitriptyline	tablet	25mg
P, S, T	634	fluoxetine	solid oral dosage form	20mg
**24.2.2. MEDICINES USED IN BIPOLAR DISODERS**				
P, S, T	635	carbamazepine	tablet (scored)	100mg
P, S, T	636	carbamazepine	tablet (scored)	200mg
P, S, T	637	carbamazepine	syrup	100mg/5ml
T	638	lithium carbonate	solid oral dosage form	300mg *200mg, 400mg
S, T	639	valproic acid (sodium valproate)	tablet (enteric coated)	200mg
S, T	640	valproic acid (sodium valproate)	tablet (enteric coated)	500mg
**24.2.3 OTHERS**				
S, T	641	olanzapine	tablets	5mg
S, T	642	olanzapine	tablets	10mg
**24.3 MEDICINES FOR ANXIETY DISORDERS**				
P, S, T	643	diazepam	tablet (scored)	2mg
P, S, T	644	diazepam	tablet (scored)	5mg
**24.4 MEDICINES USED FOR OBSESSIVE COMPULSIVE DISORDERS**				
P, S, T	645	clomipramine	capsules	10mg * tablet form

P, S, T	646	clomipramine	capsules	25mg * tablet form
**24.5 MEDICINES FOR DISORDERS DUE TO PSYCHOACTIVE SUBSTANCES USE**				
T	647	nicotine replacement therapy	chewing gum	2mg
T	648	nicotine replacement therapy	chewing gum	4mg
T	649	nicotine replacement therapy	transdermal patches	5mg to 30mg/16 hours
T	650	nicotine replacement therapy	transdermal patches	7mg to 21mg/24 hours
T	651	methadone	concentrate for oral liquid	5mg/ml
T	652	methadone	concentrate for oral liquid	10mg/ml
T	653	methadone	oral liquid	5mg/5ml
T	654	methadone	oral liquid	10mg/5ml
**Generics**	**Expanded medicinal products**	**Unregistered medicinal products (Yellow)**	**Medicinal products with slightly different specifications (Orange)**	**Unregistered medicines can be available through compounding**
**15**	**37**	**2**	**3**	**6**

**25. MEDICINES ACTING ON RESPIRATORY TRACT**				
**SECTOR**	**UID**	**MEDICINE**	**DOSAGE FORM**	**STRENGTH**
**25.1 ANTI-ASTHMATIC AND MEDICINES FOR CHRONIC OBSTRUCTIVE PULMONARY DISEASE**				
**S, T**	655	beclomethasone	inhalational (aerosol)	50mcg
**S, T**	656	beclomethasone	(Dipropionate) per dose. 100mcg	100mcg
**S, T**	658	beclomethasone	solution for nebulizers	800mcg/2ml
**S, T**	659	budesonide	inhalation (aerosol)	100mcg per dose
**S, T**	660	budesonide	inhalation (aerosol)	200mcgper dose
**P, S, T**	661	epinephrine (adrenaline)	injection	1mg in 1ml ampule
**P, S, T**	662	ipratropium bromide	inhalation (aerosol)	20mcg/metered doses
**P, S, T**	663	salbutamol	inhalation (aerosol)	100 mcg per dose
**P, S, T**	664	salbutamol	injection	50 mcg/ml in 5 ml ampule
**P, S, T**	665	salbutamol	metered dose inhaler (aerosol)	100 mcg per dose
**P, S, T**	666	salbutamol	respiratory solution for use in nebulizers	5mg/ml
**P, S, T**	667	salbutamol	tablets	100mg *2mg,4mg and 8mg
**P, S, T**	668	montelukast	sachet	4mg/sachet

**P, S, T**	669	montelukast	tablet	4mg
**P, S, T**	670	montelukast	tablet	5mg
**P, S, T**	671	montelukast	tablet	10mg
**P, S, T**	672	aminophylline	injection	25mg/ml
**P, S, T**	673	aminophylline	tablets	100mg
**P, S, T**	674	aminophylline	tablets	200mg
**P, S, T**	675	aminophylline	syrup	32mg/5ml
**Generics**	**Expanded medicinal products**	**Unregistered medicinal products (Yellow)**	**Medicinal products with slightly different specifications (Orange)**	**Unregistered medicines can be available through compounding**
7	21	1	1	0

**NEML-2018**				
**29. SPECIFIC MEDICINES FOR NEONATAL CARE**				
**SECTOR**	**UID**	**MEDICINE**	**DOSAGE FORM**	**STRENGTH**
**29.1 MEDICINES ADMINISTERED TO THE NEONATES**				
S, T	676	caffeine citrate	injection	20mg/ml
S, T	677	caffeine citrate	oral liquid	20mg/ml
P, S, T	678	chlorhexidine	solution or gel	7.1% delivering 4% chlorhexidine
P, S, T	679	ibuprofen	solution for injection	5mg/ml
S, T	680	prostaglandin E1	solution for injection	prostaglandin E 1 0.5mg/ml in alcohol *0.02mg/ml
S, T	681	prostaglandin E2	solution for injection	prostaglandin E 2 1mg/ml
S, T	682	surfactant	suspension for intratracheal instillation	25mg/ml or 80mg/ml
**29.2 MEDICINES ADMINISTERED TO THE MOTHER**				
T	683	dexamethasone	injection	4mg/ml
**Generics**	**Expanded medicinal products**	**Unregistered medicinal products (Yellow)**	**Medicinal products with slightly different specifications (Orange)**	**Unregistered medicines can be available through compounding**
**6**	**8**	**0**	**1**	**1**

S2: Category wise List of essential medicines with color-coding and result summary

NEML-2018				
30. MEDICINES FOR DISEASES OF JOINTS				
**SECTOR**	**ID NO.**	**MEDICINE**	**DOSAGE FORM**	**STRENGTH**
**30.1 MEDICINES USED TO TREAT GOUT**				
**S, T**	684	allopurinol	tablet	50mg
**30.2 DISEASE MODIFYING AGENTS USED IN PHEUMATOID ARTHRITIS**				
**S, T**	685	azathioprine	tablet	50mg
**S, T**	686	hydroxychloroquine	solid oral dosage form	200mg
**S, T**	687	methotrexate	tablet	2.5mg
**S, T**	688	penicillamine	solid oral dosage form	250mg
**S, T**	689	sulfasalazine	tablet	500mg
**30.3 JUVENILE JOINT DISEASES**				
**S, T**	690	acetylsalicylic acid	suppository	50mg to 150mg
**S, T**	691	acetylsalicylic acid	tablet	100mg to 500mg
**Generics**	**Expanded medicinal products**	**Unregistered medicinal products (Yellow)**	**Medicinal products with slightly different specifications (Orange)**	**Unregistered medicines can be available through compounding**
**7**	**8**	**1**	**0**	**1**
