# Supplementary material for: Availability of essential medicines in Pakistan—A comprehensive document analysis
Source: PLoS One. 2021 Jul 9;16(7):e0253880. doi: 10.1371/journal.pone.0253880 (PMC8270130; doi:10.1371/journal.pone.0253880)
Supplement: S3 File — (PDF) [file pone.0253880.s003.pdf]

S3						
**S3 File: List of medicines with multiple appearance in NEMLPK-2018**						
	**First appearance**		**Second appearance**		**Third appearance**	
No.	**UID**	**Medicine Description**	**UID**	**Medicine Description**	**UID**	**Medicine Description**
1	19	Inj. atropine 1mg in 1ml ampoule	97	Inj. atropine 1mg in 1ml ampoule		
2	24	Suppository acetylsalicylic acid 50mg-150mg	690	Suppository acetylsalicylic acid 50mg-150mg		
3	25	Tab. acetylsalicylic acid (aspirin) 75mg EC	691	Tab. acetylsalicylic acid (aspirin) 75mg EC		
4	71	Tab. bisacodyl 5mg	556	Tab. bisacodyl 5mg		
5	47	Inj. dexamethasone 4mg/ml	79	Inj. dexamethasone 4mg/ml	683	Inj. dexamethasone 4mg/ml
6	53	Rectal soln. diazepam 2.5mg	108	Rectal soln. diazepam 2.5mg		
7	55	Rectal soln. diazepam 10mg	109	Rectal soln. diazepam 10mg		
8	57	Tab. diazepam 10mg	644	Tab. diazepam 10mg		
9	58	Inj. haloperidol 5mg in 1ml ampoule	625	Inj. haloperidol 5mg in 1ml ampoule		
10	59	Oral liq. haloperidol 2mg/ml	626	Oral liq. haloperidol 2mg/ml		
11	60	Tab. haloperidol 0.5mg	627	Tab. haloperidol 0.5mg		
12	61	Tab. haloperidol 2mg	628	Tab. haloperidol 2mg		
13	62	Tab. haloperidol 5mg	629	Tab. haloperidol 5mg		
14	80	Inj. epinephrine (adrenaline) 1mg/1ml ampoule	661	Inj. epinephrine (adrenaline) 1mg/1ml ampoule		
15	103	Oral liq. carbamazepine 100mg/5ml	635	Oral liq. carbamazepine 100mg/5ml		
16	106	Tab. (scored) carbamazepine 100mg	636	Tab. (scored) carbamazepine 100mg		
17	123	Tab. (enteric coated) valproic acid (sodium valproate) 200mg	639	Tab. (enteric coated) valproic acid (sodium valproate) 200mg		
18	124	Tab. (enteric coated) valproic acid (sodium valproate) 500mg	640	Tab. (enteric coated) valproic acid (sodium valproate) 500mg		

19	107	Tab. (scored) carbamazepine 200mg	637	Tab. (scored) carbamazepine 200mg		
20	188	Inj. amikacin sulfate 100mg	255	Inj. amikacin sulfate 100mg		
21	194	Susp. doxycycline 25mg/5ml	209	Susp. doxycycline 25mg/5ml		
22	195	Susp. doxycycline 50mg/5ml	210	Susp. doxycycline 50mg/5ml		
23	196	Cap. doxycycline 50mg	211	Cap. doxycycline 50mg		
24	197	Cap. doxycycline 100mg	212	Cap. doxycycline 100mg	376	Cap. doxycycline 100mg
25	215	Inj. metronidazole 500mg in 100ml vial	354	Inj. metronidazole 500mg in 100ml vial		
26	216	Susp. metronidazole 200mg/5ml	355	Susp. metronidazole 200mg/5ml		
27	217	Tab. metronidazole 400mg	356	Tab. metronidazole 400mg		
28	329	Inj. ribavirin for I/V administration 800mg in 10ml phosphate buffer solution	343	Inj. ribavirin for I/V administration 800mg in 10ml phosphate buffer solution		
29	330	Inj. ribavirin for I/V administration 1g in 10ml phosphate buffer solution	344	Inj. ribavirin for I/V administration 1g in 10ml phosphate buffer solution		
30	434	Tab. propanalol 10mg	459	Tab. propanalol 10mg		
31	435	Tab. propanalol 40mg	460	Tab. propanalol 40mg		
32	436	Tab. propanalol 80mg	461	Tab. propanalol 80mg		
33	437	Tab. verapamil 40mg	448	Tab. verapamil 40mg		
34	438	Tab. verapamil 80mg	449	Tab. verapamil 80mg		
35	439	Tab. bisoprolol 1.25mg	457	Tab. bisoprolol 1.25mg	476	Tab. bisoprolol 1.25mg
36	440	Tab. bisoprolol 5mg	458	Tab. bisoprolol 5mg	477	Tab. bisoprolol 5mg
37	462	Inj. hydralazine (HCl) 20mg	465	Inj. hydralazine (HCl) 20mg		
38	463	Tab. enalapril 2.5mg	482	Tab. enalapril 2.5mg		
39	464	Tab. enalapril 5mg	483	Tab. enalapril 5mg		
40	468	Oral liq. Hydrochlorothiazide 50mg/5ml	482	Oral liq. Hydrochlorothiazide 50mg/5ml		
41	470	Tab. hydrochlorothiazide 25mg	483	Tab. hydrochlorothiazide 25mg	513	Tab. hydrochlorothiazide 25mg

42	471	Inj. hydrochlorothiazide 20mg	514	Inj. hydrochlorothiazide 20mg		
43	489	Tab. spironolactone 25mg	520	Tab. spironolactone 25mg		
44	485	Oral liq. Frusemide 20mg/5l	509	Oral liq. Frusemide 20mg/5l		
45	486	Tab. furosemide 40mg	512	Tab. furosemide 40mg		
46	549	Tab. sulfasalazine 500mg	689	Tab. sulfasalazine 500mg		
