# Supplementary material for: Availability of essential medicines in Pakistan—A comprehensive document analysis
Source: PLoS One. 2021 Jul 9;16(7):e0253880. doi: 10.1371/journal.pone.0253880 (PMC8270130; doi:10.1371/journal.pone.0253880)
Supplement: S4 File — (PDF) [file pone.0253880.s004.pdf]

S4 File: List of incomplete information, missing strength, wrong specifications, and typographic mistakes

Status	Therapeutic Category	UID	Generic	Dosage Form in 7th NEML of Pakistan 2018	Dosage form in 20th WHO model list of essential medicines
Registered	Anesthetics	1	isoflurane	Inhalational	Inhalational
Registered	Anesthetics	2	sevoflurane	Inhalational	Inhalational
Medical gases: Do-not need registration status yet	Anesthetics	3	nitrous oxide	Inhalational	Inhalational
Medical gases: Do-not need registration status yet	Anesthetics	4	oxygen	inhalational (medicinal gas)	inhalational (medicinal gas)
Slightly different specifications	Anti-dotes and other substances used in poisoning	93	activated charcoal	Powder	Powder
Wrong specifications/typographic mistake	Anti-infectives	153	Ampicillin 125 mg/ml* (125 mg/5 ml)	Syrup	Not included
Wrong specifications/typographic mistake	Anti-infectives	154	Ampicillin 250 mg/ml* (250 mg/5 ml)	Syrup	Not included
Dispensing item	Diuretics	521	sodium polystyrene sulfonate	Powder	Not included
Dispensing item	Gastro-intestinal medicines	522	pancreatic enzymes	Age-appropriate formulation	Age-appropriate formulation
Consumer item: Do not need registration status	Hormones, other endocrine medicines and contraceptives	576	Copper containing devices		
Consumer item: Do not need registration status	Hormones, other endocrine medicines and contraceptives	578	condoms		
Consumer item: Do not need registration status	Hormones, other endocrine medicines, and contraceptives	579	diaphragm		
Unregistered	Hormones, other endocrine medicines, and contraceptives	583	estrogen		
Registered	Muscle relaxants	607	suxamethonium	powder for injection	
Wrong specifications	Medicines acting on respiratory tract	667	salbutamol 100 mg* 2mg, 4mg, 8mg	Tablets	Not included

**wrong specification*
